# Efficient and accurate detection of splice junctions from RNA-seq with Portcullis

**DOI:** 10.1093/gigascience/giy131

**Published:** 2018-12-12

**Authors:** Daniel Mapleson, Luca Venturini, Gemy Kaithakottil, David Swarbreck

**Affiliations:** Earlham Institute, Norwich Research Park, NR47UZ, Norwich, United Kingdom

**Keywords:** splice junction, RNA-Seq, BAM

## Abstract

Next-generation sequencing technologies enable rapid and cheap genome-wide transcriptome analysis, providing vital information about gene structure, transcript expression, and alternative splicing. Key to this is the accurate identification of exon-exon junctions from RNA sequenced (RNA-seq) reads. A number of RNA-seq aligners capable of splitting reads across these splice junctions (SJs) have been developed; however, it has been shown that while they correctly identify most genuine SJs available in a given sample, they also often produce large numbers of incorrect SJs. Here, we describe the extent of this problem using popular RNA-seq mapping tools and present a new method, called Portcullis, to rapidly filter false SJs derived from spliced alignments. We show that Portcullis distinguishes between genuine and false-positive junctions to a high degree of accuracy across different species, samples, expression levels, error profiles, and read lengths. Portcullis is portable, efficient, and, to our knowledge, currently the only SJ prediction tool that reliably scales for use with large RNA-seq datasets and large, highly fragmented genomes, while delivering accurate SJs.

## Introduction

Alternative splicing (AS) is a regulated process in Eukaryotic species that occurs during gene expression, enabling a single gene to code for multiple proteins through inclusion or exclusion of exons in the transcribed mRNA. Key to defining the complexity of alternative splicing within a gene is the identification of splice junctions (SJs), which occur at exon-exon boundaries and are typically characterized in pairs representing both the donor site (5’ intron boundary to 3’ upstream exon boundary) and acceptor site (3’ intron boundary to 5’ downstream exon boundary). A recent study has given us a comprehensive view into alternative splicing in humans [1], although annotations of other model species are known to be incomplete [2]. This lack of completeness reduces the accuracy and usefulness of many downstream gene and transcript level tasks, such as differential expression analysis [3] and alternative splicing analysis [4].

RNA sequencing (RNA-seq) has become the standard method to detect, quantify, compare, and contrast splice isoforms across different biological contexts [5]. Furthermore, as next-generation sequencing (NGS) technologies have matured, RNA-seq is becoming increasingly quick, reliable, and cost-effective [6]. SJs derived from RNA-seq studies are primarily detected via splice-aware mapping tools, which can split RNA-seq reads across introns in the genome. However, a survey of many RNA-seq mapping tools highlighted that accurate detection of SJs is an outstanding challenge [7]. Here, we show that this issue persists with recent versions of many popular RNA-seq mappers. The lack of accuracy is due to various factors, such as short read lengths that increase mapping ambiguity and sequencing errors that trigger misaligned split reads. The problem is exacerbated in deeply covered datasets where the likelihood of generating distinct invalid splice sites increases.

There are a number of strategies for reducing the number of distinct spurious SJs found in mapped reads. One method involves counting the split reads supporting each distinct junction and filtering those under a certain threshold [1]. Other methods utilize the length of split read overhangs across each junction from the set of supporting split reads [8] or calculate the amount of evidence supporting their start sites [9]. Studying the genome around the SJ has also proven effective [10–12]. To reduce the propagation of invalid junctions into downstream tasks, many RNA-seq mappers offer the ability to use a set of high-confidence junctions to guide realignment of the reads.

In practice, the computational demands of collecting a set of accurate junctions rapidly can be problematic, often leading to compromised accuracy. Accurate methods require long run imes, high-memory usage, or are inflexible and difficult to use. We address these issues with our tool, Portcullis, which is the only method we are aware of for rapidly and accurately filtering invalid SJs from binary alignment map (BAM) alignments produced by any RNA-seq mapper. Over a wide range of scenarios, we show that Portcullis competes with, or outperforms, the best methods currently available in terms of accuracy, speed, and memory efficiency. Portcullis also offers rich junction analysis and quantification capabilities, as well as a supplementary tool kit, called Junctools, for performing tasks such as junction file format conversions and junction set comparisons and for providing various junction and transcript filtering options.

## Results

### Junction detection performance

It has been observed that short-read RNA-seq mapping tools often produce large numbers of false-positive junctions [7]. To gauge the extent of this problem with a more recent set of popular mapping tools, we generated several sets of simulated reads with varying read lengths and depths from three different model species’ transcriptomes. We then extracted a distinct set of SJs from split reads produced by the mappers and compared them to the set of true junctions for each corresponding simulated dataset. As it is impractical to derive a comprehensive set of false junctions,^1^ we use the recall, precision, and *F*_1_ (F-measure) [13] to assess the performance of each mapper (see Supplementary Section 2 for more information).

**Figure 1:**
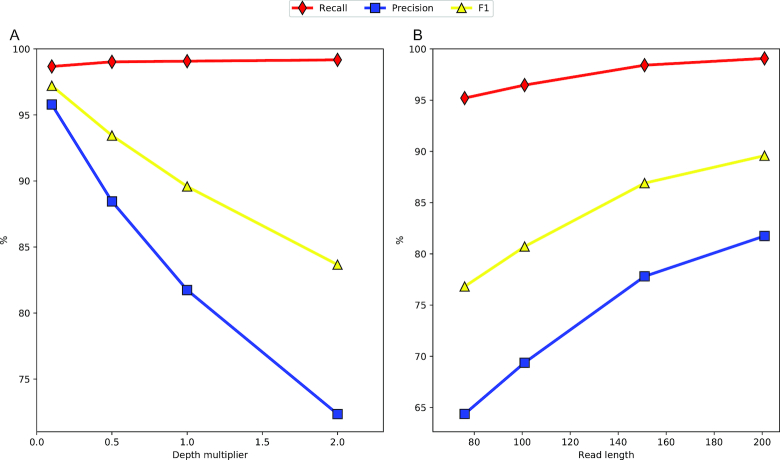
Splice junction accuracy of STAR v2.6.0a across variations of our simulated Human dataset. **(A)** Scatter plot showing the effect of varying dataset size, with all datasets containing 201 bp reads. The 1.0X depth multiplier represents a dataset of ∼78 million read pairs. **(B)** Scatter plot showing the effect of varying read length with all datasets containing ∼30 billion base pairs.

**Figure 2:**
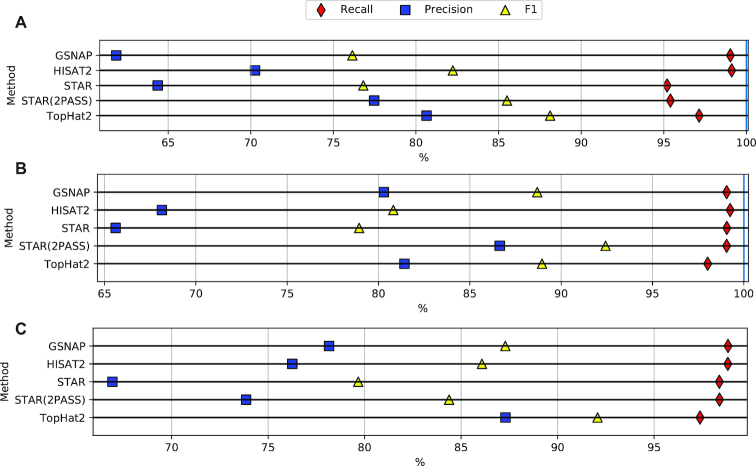
Splice junction detection performance across mappers for 76 bp simulated paired reads. The Human dataset **(A)** contains 421,020,756 reads across 19,853 transcripts. The *Arabidopsis* dataset **(B)** contains 148,207,902 reads across 19,723 transcripts. The *Drosophila* dataset **(C)** contains 202,246,654 reads across 9,376 transcripts.

Figure 1A shows the performance of STAR v2.6.0a for four 201 bp paired-end read datasets of the Human transcriptome, each with varying depth. Figure 1B shows the effect of varying read length, with each dataset having a depth of ∼30 billion base pairs. The plots show that a longer read length improves both recall and precision; however, increased depth, while marginally improving recall, decreases precision significantly, pulling *F*_1_ down with it. This precision decrease is in part attributable to reads containing sequencing errors triggering misalignments of split reads, leading to new, invalid junctions being predicted [9]. Supplementary Section 5 shows that the same trends hold true for several other popular RNA-seq mappers such as TopHat2 v2.1.0 [14], GSNAP v20180530 [15], and HISAT v2.1.0 [16]. In addition, Fig. 2 highlights that the effect is not limited to Human, a species with complex splicing behavior, but is also visible in species displaying significantly less splicing events, such as *Arabidopsis* and *Drosophila* .

**Figure 3:**
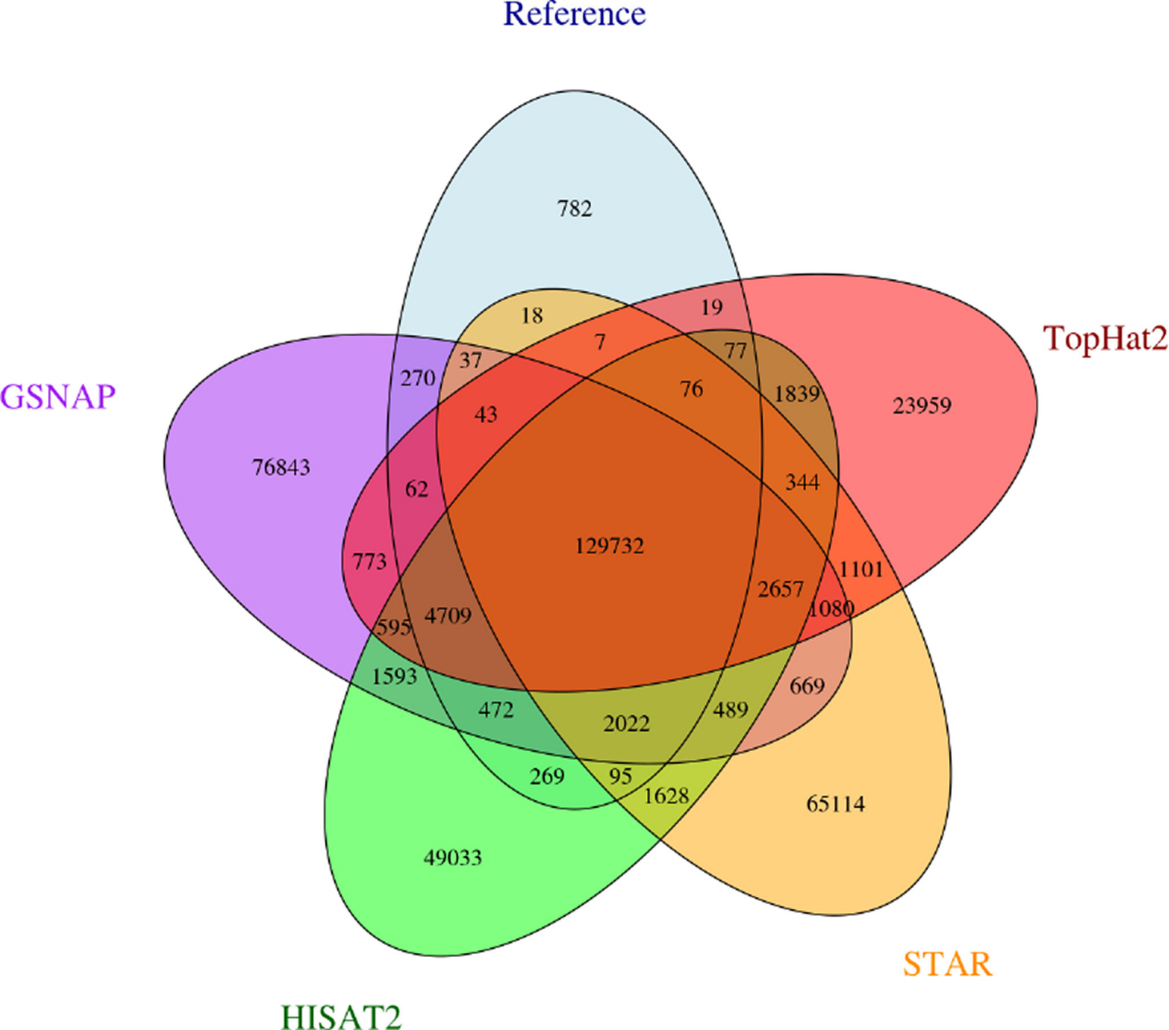
A five-way Venn diagram showing levels of agreement between mapping tools and the Human junction truth set with 76 bp simulated reads..

We also observed that while all of these mappers recall a good fraction of genuine junctions, the mappers are not producing the same sets of false positives. The Venn diagram in Fig. 3 shows the agreement between junctions found across Tophat2, GSNAP, STAR, and HISAT2 mappers on our 76 bp simulated Human dataset. Each mapper retrieves at least 95.2% of true junctions, although there is little agreement for the remaining junctions, indicating that these mappers often make different types of mistakes. Supplementary Fig. S4 shows the effect of different depth and read length, but essentially the same trends hold. These observations indicate that a high-confidence set of SJs can be built by requiring a degree of concordance between multiple mappers, which we demonstrate in Supplementary Fig. S5. As the expected number of mappers required to agree increases, there is a corresponding increase in precision at the expense of recall. We note that in the cases when 2, 3, or 4 mappers are required to agree, the *F*_1_ exceeds that of any of the individual mapping tools. However, this approach has several disadvantages. First, it assumes that the aligners find different false positives.^2^ Second, a single poorly performing mapper will reduce the performance of the system as a whole. Third, it is not possible to know *a priori* how much agreement is required to get optimal results. Finally, this approach is not computationally efficient as it requires running multiple tools for a single dataset.

An alternative approach is to analyze the set of mapped split reads supporting each SJ to produce a set of metrics around how those reads stack up around the SJ and then apply some criteria to determine if that SJ is likely to be genuine or invalid. This can involve using rules based on some pre-defined cutoff values (e.g., discard SJs supported by less than five reads); however, only modest gains can be achieved this way, as we show in Supplementary Section 3. Better results are gained through a more comprehensive analysis, combining multiple metrics, including metrics derived from analysis of the genome at splice site locations, which typically come at the expense of time and computational resources. Some tools that do this include FineSplice v0.2.2 [12], TrueSight v0.06 [11], MapSplice v2.2.1 [9], and SOAPsplice v1.10 [10]. These methods vary in their implementation and intended usage. MapSplice, TrueSight, and SOAPsplice are stand-alone tools designed to be used as replacements for RNA-seq mapping tools. They process reads directly and produce alignments and lists of detected junctions. Finesplice is a post-alignment junction filtering tool, requiring a TopHat2 BAM file as input, which produces a list of filtered SJs as its final output.

However, as we will show, all of these tools have specific disadvantages, which prompted us to develop our own method, Portcullis. Portcullis has an architecture that is similar to that of Finesplice, consuming BAM files as input and producing a set of filtered SJs as output. However, Portcullis is distinct from Finesplice in that it also produces an analysis of all SJs present in the input BAM and can consume BAMs generated from any RNA-seq mapper, not just TopHat2.

**Figure 4:**
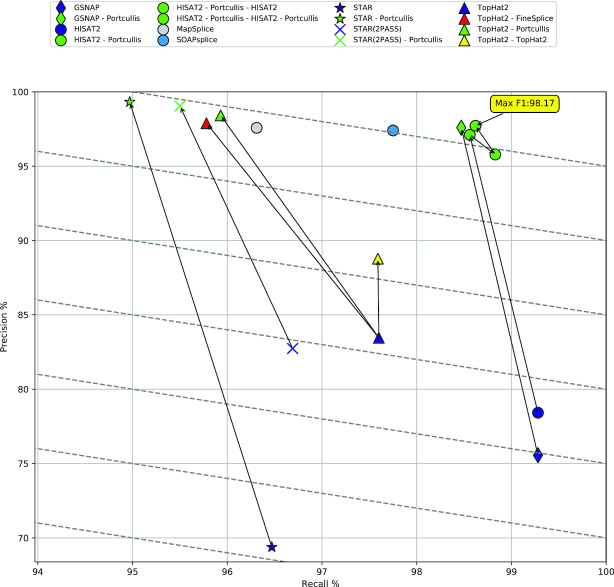
A scatter plot showing recall and precision results of all methods on our 101 bp ∼76 million simulated Human read dataset. Diagonal lines represent actual *F*_1_ score gradients. Arrows show the effect of processing BAM files by downstream junction filtering tools such as Portcullis or FineSplice. The purple TopHat2 entry shows the effect of TopHat2’s own rule-based filtering on the BAM file.

We undertook an experiment to observe how these methods perform when varying sequencing depth, read length, and species. The full results are presented in Supplementary Figs. 6-8, although we provide a recall/precision scatter plot in Fig. 4 that summarizes many of the same points in a single plot. This plot shows how the methods compare when run on our 101 bp simulated Human dataset. The difference between the more accurate methods and the original RNA-seq mappers is stark, with precision scores not dropping below 97% compared to precision lower than 85% in all cases for the mappers. Because Finesplice and Portcullis are dependent on the alignments produced by RNA-seq mapping tools, it is impossible for them to outperform their input in terms of recall, so their goal is to discard invalid junctions but not genuine ones. Despite a small drop in recall, Portcullis significantly improves the precision of SJ calls over all input methods, producing higher *F*_1_ scores overall and outperforming FineSplice on TopHat2 input. In addition, when coupled with GSNAP or HISAT2, Portcullis produces the highest overall *F*_1_ scores of any method on this dataset. For most methods, increasing read length improves results, although while SOAPsplice has a comparable *F*_1_ score in this plot, Supplementary Fig. S6 shows that increasing read length over 101 bp decreases SOAPsplice’s accuracy drastically. Furthermore, we can make additional minor improvements to *F*_1_ by running the RNA-seq mappers in a two-pass configuration, feeding in Portcullis-predicted junctions in the second pass to be used as a guide during the alignment process. The effect of applying this strategy with HISAT2 is shown in Fig. 4 (upper right corner), and it could be similarly applied with other mappers. This has the additional benefit of generating a Sequence Alignment Map (SAM)/BAM file with more accurate alignments around SJs, which is useful for downstream analysis, coming at the expense of additional runtime requirements.

**Figure 5:**
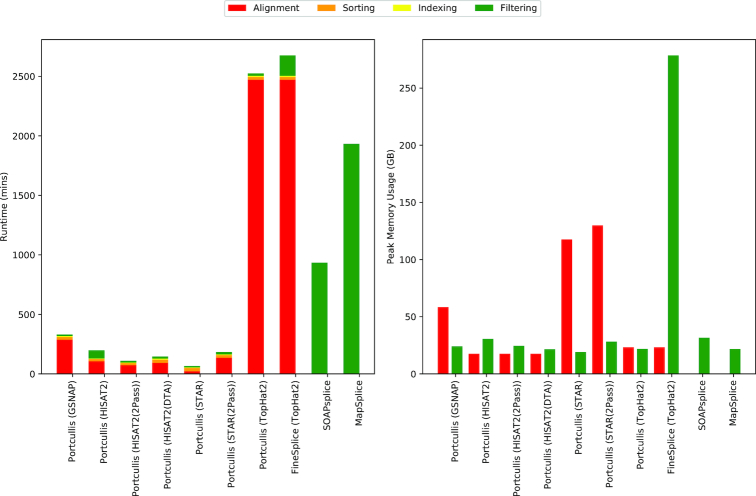
Runtimes and max memory usage of all methods on our 101 bp ∼76 million simulated Human read dataset using eight threads where appropriate. For FineSplice and Portcullis, times for alignment, sorting, and indexing are factored into the results. For memory usage, we consider alignment and filtering stages only.

The runtimes and max memory usage associated with methods that deliver high accuracy in Fig. 4 are shown in Fig. 5, with results across all datasets shown in Supplementary Figs. S10 and S11. The results include time taken to align, sort, and index the input reads where required, in addition to filtering the junctions. All methods were run with eight threads where possible, and we would also like to point out that Portcullis’ memory usage can be lowered further by reducing the number of threads, as shown in Supplementary Fig. 12.

This runtime analysis immediately highlights issues with several methods. First, TopHat2 suffers from long runtimes, which should be considered when running downstream filtering tools. This is especially problematic to FineSplice, which can only run on TopHat2 alignments. FineSplice also suffers from high memory usage, which grows quickly with the number of reads present in the dataset. High memory usage is also an issue with GSNAP, STAR, and TrueSight. Indeed, we terminated the TrueSight job on our 101 bp Human dataset after a week of processing. Both SOAPsplice and MapSplice have relatively long runtimes compared to Portcullis coupled with GSNAP, STAR, or HISAT2. Portcullis performs well, both in terms of accuracy and practicality, across varying sequencing depth, read lengths, and species, particularly when coupled to HISAT2.

### Analysis of real data

RNA-seq simulations, while useful for benchmarking methods, do not give a full description of the complexity and noise inherent in real data. For example, our artificial datasets only simulated transcripts present in the reference annotations, which are heavily biased towards coding transcripts [17]. Real data will contain many more non-coding elements and may contain other biological entities that have not been properly annotated yet.

We predict that Portcullis is more useful on real data than on simulated data, given that it appears to have a greater effect on noisier and deeper datasets. However, comparing the accuracy of methods on real data is more challenging for several reasons. Firstly, even for model organisms, the reference annotations are incomplete and RNA-seq experiments will likely contain some genuine novel junctions, which appear as false positives, meaning precision scores are unreliable. Furthermore, when comparing precision scores between methods, it does not necessarily follow that higher precision is a better result, as this could be interpreted as the tool is worse at finding lower confidence junctions. Secondly, a single RNA-seq experiment is unlikely to cover all junctions found in the reference, meaning that it is impossible to achieve perfect recall, although typically we can compare recall scores between methods, as a higher number of correctly detected reference junctions suggests that the method is genuinely more sensitive.

To get a better feel for the validity of SJs on real data, it is helpful to take a finer-grained approach. For simulated data, SJs were considered as pairs, representing both ends of the intron, and that SJ pair is considered genuine only if both SJs are found in the reference. Instead of considering just two classes (genuine and invalid) for this pairing, it is possible to break down each intron into four distinct classes, each with decreasing likelihood of representing a genuine SJ [11]:

1. introns matching annotated known introns (i.e., both splice sites match the same intron in the reference)
2. introns with donor and acceptor sites present in annotation but matching different introns
3. introns with only one annotated splice site (i.e., only one splice site found in the reference)
4. introns with two novel splice sites (i.e., both not found in the reference)

By classifying junctions this way, the precision of methods can be compared based on higher counts as the class number increases. To demonstrate this point, we extracted junctions from our simulated 76 bp Human dataset and compared them against the real Human reference. The results shown in Supplementary Fig. S13 indicate the majority of junctions occurring in class 1, with similar counts across methods. This is expected for simulated data as all correct junctions should only be found in class 1, with false positives seen by high counts in classes 2, 3, and 4.

**Figure 6:**
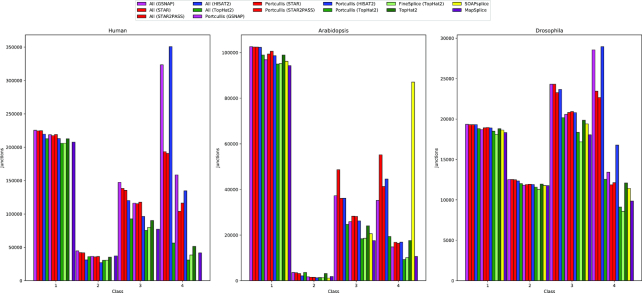
In this plot, we check junctions found via each method against the reference annotation for Human (251 bp reads), Arabidopsis (151 bp reads), and Drosophila (101 bp reads), respectively. The results are categorized into the following classes: intron match; both splice sites found; one splice site found; and no splice sites found. SOAPsplice did not finish for the Human dataset, and TrueSight failed to finish successfully on all datasets due to memory demands.

To assess the performance of Portcullis on real data, we used the same real datasets that provided error models for our simulated dataset and checked junctions predicted by various methods against the reference annotation for each species: Human, Arabidopsis, and Drosophila. The results shown in Fig. 6 indicate that counts in class 1 have little variation between methods. This implies that the methods that perform well on simulated data do not discard many genuine junctions incorrectly. As expected, counts in class 4 have a relatively high variation between methods across species. The methods identified as being most imprecise for our simulated data also have higher counts in this class relative to the more precise methods. This suggests that much of the difference is likely explained by false positives. For Portcullis, the large drop between input mappers and Portcullis results is reassuring and suggests that Portcullis’ predictions in this class are likely to contain a higher proportion of genuine novel junctions than the input. These plots again highlight SOAPsplice’s difficulty processing longer reads. As for the Human dataset (251 bp), SOAPsplice failed completely; with Arabidopsis (151 bp), we can see a very high number of class 4 junctions. This is in contrast to the Drosophila dataset, which contains 101 bp reads where SOAPsplice appears to perform well and is in line with counts from Portcullis and MapSplice. We could not get TrueSight to run on any of these real datasets due to excessive memory runtime requirements.

## Use case for non-model organisms

**Figure 7:**
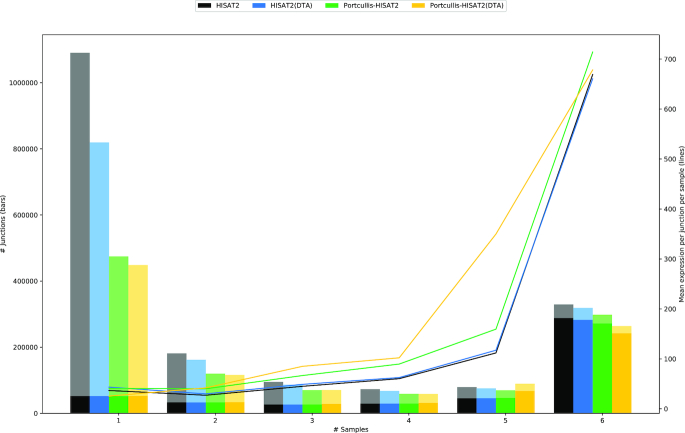
Junction counts that are supported by one through six samples for wheat RNA-seq data. Solid tint indicates that junctions were found in the reference annotation; paler tint indicates junctions were not found in the reference. Junctions that occur in all six samples are more likely to be found in the reference. Average expression per junction per sample is shown by the lines and indicates that junctions found in all samples have high expression.

To demonstrate the use of Portcullis in a challenging real-life scenario, we created HISAT2 alignments for six Chinese spring wheat RNA-seq samples [18], producing 1,515,705,216 alignments of 251 bp reads across all datasets. We aligned HISAT2 with and without HISAT2’s Downstream Transcript Assembly (DTA) mode, which is intended to reduce the number of false-positive SJs in the aligned data. We then ran Portcullis on each set of aligned reads. Using Junctools (see Supplementary Section 6), we took the union of junctions across each set of samples, marking up the number of samples each junction occurs in, along with whether the junction can be found in the wheat reference annotation. Figure 7 shows that junctions found in all samples are also likely to be found in the reference annotation and have a high expression. Conversely, junctions detected in a single sample have a lower expression on average, meaning they are less likely to be incorporated into the reference annotation and are more likely to be false positives.

The overall number of junctions for Portcullis in both modes is similar: 1,091,198 for HISAT2 and 1,047,335 for HISAT2(DTA), whereas the difference in the number of junctions found in the pre-filtered alignments was much larger: 1,848,057 and 1,529,999, respectively. Furthermore, the number of annotated junctions found in a single sample are consistent across all datasets, suggesting that Portcullis is not falsely discarding many junctions that are likely to be genuine.

To demonstrate Portcullis’ ability to handle large datasets, we merged the BAMs from all six samples, producing a single BAM containing alignments of ∼755 million 251 bp reads. Portcullis processed these in 400 minutes, using four threads and requiring a peak of 59 GB of RAM on a machine with 4 AMD Opteron(tm) 6134 processors. The full set of HISAT2 alignments consisted of 1,845,781 junctions, with 1,092,390 junctions remaining after filtering. This figure is very close to the result gained by filtering each sample individually and then taking the union.

## Discussion

Here, we confirmed that recent versions of several popular RNA-seq alignment tools still suffer from high numbers of false-positive SJs in their output. Achieving the best results requires a more thorough and computationally intensive analysis of alignments around splice sites and interrogation of the genome at these loci. However, most other tools that achieve high accuracy are either impractical or unreliable across different datasets and species. Portcullis is the only junction prediction tool that achieves these results while scaling to the requirements of modern NGS workflows. We demonstrated that Portcullis is capable of analyzing a wheat dataset comprising ∼755 million 251 bp reads merged from six separate samples on a fragmented version of the genome containing 735,943 contigs. Portcullis ran to completion in 400 minutes using four threads with less than 60 GB of RAM, making it feasible to process extremely large, complex datasets with readily available hardware.

Apart from high accuracy and efficient runtime performance, we see that Portcullis also has significant advantages over other methods in its flexibility and ease of use. Especially notable is the fact that it completely decouples junction filtering from RNA-seq mapping, enabling the user to select the mapper of their choice, should more attractive mapping options be available in the future.

In addition, Portcullis’ supplementary toolkit, Junctools, ensures that Portcullis’ output is easy to incorporate into workflows that use other tools, reducing the amount of custom scripting required by a bioinformatician. Junctools also makes it straightforward to integrate Portcullis into a two-pass alignment approach, whereby Portcullis junctions are converted to a format suitable for a particular aligner via Junctools, then used as a guide to produce more accurate alignments. In particular, we see that coupling HISAT2 with Portcullis in two-pass mode delivers high accuracy within a reasonable time frame and acceptable memory usage.

Accurate SJ prediction allows us to get richer and more useful information from downstream tasks such as alternative splicing analysis. Each missed SJ reduces the richness of the transcript model, and each false positive can lead to incorrect donors, acceptors, and cassettes. This is particularly important in non-model organisms with incomplete annotations. In addition, transcript assemblies could be improved either by filtering transcripts containing unsupported SJs (as implemented in Mikado [19]) or by producing more accurate input reads via the two-pass approach mentioned previously. Portcullis junctions can be used to provide additional hints to gene prediction tools to help select between sets of alternative isoforms. This way we envisage Portcullis assisting the production of both richer and more precise genome annotations for Eukaryotic organisms.

## Materials and Methods

### Simulation of RNA-seq data

To compare performance between junction filtering tools, we created several simulated RNA-seq datasets based on three known model organisms (Human, Drosophila, and Arabidopsis), with error and expression profiles derived from real datasets using SPANKIsim [20]. This produces reads derived from a known region in the reference transcriptome, along with the perfect alignments of those reads. From the alignments it is possible to unambiguously derive the true set of junctions for the given dataset, providing a platform from which RNA-seq mappers and SJ filtering tools can be benchmarked. The complete pipeline used to generate the simulated reads is described in Supplementary Section 1.1. Basic statistics for the datasets are described in Table 1, reference annotations were obtained from Ensembl [28] and The Arabidopsis Information Resource (TAIR)[29].

**Table 1.** Properties of core simulated datasets for each species

Properties of simulated dataset	Arabidopsis	Drosophila	Human
Reference annotation	TAIR10	DMEL78	HG38
Original accession	PRJEB7093	SRA009364	PRJEB4208
Millions of reads (in original)	93	47	97
Mean quality (in original)	37	37	39
Depth fractions	10%, 50%,100%	10%, 50%,100%	10%, 50%,100%, 200%
Millions of paired reads	9,47,93	5,24,47	7,38,76,152
Read lengths @ 100% depth (bp)	76,101	76	76,101,151,201
Number of splice junctions @ max readlen and 100% depth	90,190	29,275	139,403
% of SJ’s in ref	71	51	42
Number of transcripts @ max readlen and 100% depth	19723	9376	19853
% of transcripts in ref	47	32	12

### Portcullis

**Figure 8:**
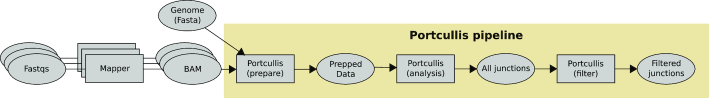
A high-level view of the Portcullis pipeline. Input to Portcullis is a genome in FastA format and one or more BAM files created by an upstream RNA-seq mapping tool. The first stage ensures the alignments are correctly merged, sorted, and indexed, then all junctions found in the input are analyzed and output to disk. Next, the full set of junctions is filtered to remove likely false positives and also output to disk. The user can choose to either run the full pipeline in one go or at each stage separately.

The pipeline for Portcullis, shown in Fig. 8, describes the flow of data from sequenced RNA reads in FastQ format through to a set of filtered junctions provided by Portcullis. RNA-seq files must first be mapped with a suitable RNA-seq mapper and converted into BAM format. Portcullis takes in one or more BAM files and a genome in FastA format as input. The first stage in Portcullis ensures all input data are prepared in a way suitable for downstream processing. This includes if multiple BAM files are provided merging them into a single file, then ensuring that the BAM is coordinate sorted, and that both the BAM and genome file are properly indexed. All distinct junctions are extracted from the BAM file and analyzed, which are then filtered, keeping only the high-quality junctions. The output is both the full set and the filtered set of junctions in both a descriptive tabular format and an exon-based bed format.

#### Junction analysis

Portcullis identifies all split reads in SAM/BAM format through reference skipping cigar operations ("N"). All split reads are then collapsed based on the locations of reference skipping regions into a distinct set of potential junctions. Portcullis then makes observations about each potential junction based on the RNA-seq data and genomic features at those loci. A definitive list of features is described with the software’s documentation, although we describe a few of the more important ones here.

First, we provide a number of ways to quantify each SJ. This includes the raw count of the number of split reads supporting the junction, the count of split reads containing only a single intron (uniquely split reads), as well as how many are uniquely mapped (uniquely mapped split reads). In addition, we record the number of split reads deemed to be "reliable," which we define a uniquely mapping and properly paired. Furthermore, we find that that junctions possessing high ratios of reliable split reads to the total number of raw split reads is a useful indicator of junction quality.

Assuming random sampling of sequenced reads, it is expected that the start sites of split reads will be uniformly distributed across the upstream anchor of the junction. This notion is captured in the Shannon entropy score [9]. Junctions with a high number of split reads can therefore have a low entropy score if those reads start at a small number of sites and are therefore less likely to be genuine. Similarly, junctions with a moderate number of supporting reads can have high entropy (and therefore are more likely to be genuine) if many of them have distinct start sites. This concept is extended further to show the deviation from expected to observed read counts at each anchor position [12], providing a more detailed picture across the split read overhangs up- and downstream of the intron. Typically, we expect a gradual reduction of coverage across the length of the junction (up to half the read length). Where we see sharp deviations from this, the chance of an invalid junction increases.

Another frequently used approach is to calculate the maximal split read overhang, a method used in both TopHat2 and STAR for filtering junctions. The best score possible for a given junction is the maximum split read length divided by 2. A more sophisticated version of this concept includes modifying this score by penalizing alignments containing mismatches. This approach is called the maximum of the minimal match on either side of the SJ (MaxMMES) [8].

**Figure 9:**
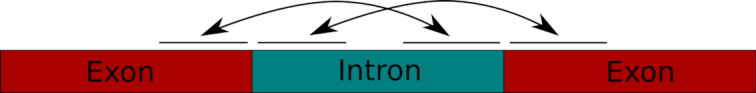
Calculating the hamming distinct of both the right-most region of the left anchor to the right-most region of the intron and the left-most region of the intron to the left-most region of the right anchor can give an indication of whether the splice site may have been incorrectly triggered by a repeat region in the genome.

In terms of genomic information, we consider the composition of the two-base donor and acceptor sites, which in most, but not all, cases conform to the same canonical, or semi-canonical, pattern [21]. We also look at hamming distances between the left anchor and right side of the intron, as well as the left intron and right anchor. This provides an indication of whether the splice sites are embedded in a repeat region [20] and therefore unlikely to be genuine. This idea is illustrated in Fig. 9.

In Supplementary Section 3 we show that it is possible to collect precise subsets of genuine and invalid SJs across different species and datasets through aggressive rule-based filtering. These subsets can be leveraged to extract information that allows additional features to be calculated for all junctions. One such feature is an intron size score, which must adapt to significant variation in average length between species [22]. This metric is based on the assumption that SJs with excessively long introns (adjusted for the given species) are likely to be incorrect. Junctions with an intron size less than the intron size at the 95th percentile in the positive set have a score of 0 and those above are assigned a positive score [11]. Another feature is a score used to represent the strength of the splicing signal based on probabilities of nucleotide frequencies at positions in the genome around the donor and acceptor sites [11]; this is an idea that has previously been exploited in *ab initio* gene prediction tools [23].

While supplying additional features can improve predictive performance, sometimes if not enough examples are provided, it increases the chance a classifier can overfit to the training set [24]. Additional redundant features will also slow down training times. We attempted to engineer our features to balance accuracy (both training set accuracy as well as validation set accuracy) and runtime by discarding features determined to not be useful for classification purposes. We achieved this by running our classifier through permutations of features and removing those that had no, or a negative, change in accuracy across all our simulated datasets and repeated this process until no further gains we apparent. The features that we utilize in our model are:

- Reliable reads
- Reliable to raw read ratio
- MaxMMES
- Mean mismatches per read
- Intron score
- Min hamming score
- Position weight matrix
- Splicing signal
- Deviation of expected to observed read counts at anchor positions (up to 20)

#### Filtering junctions

**Figure 10:**
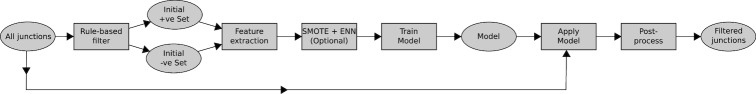
An exploded view of the Portcullis filtering stage. Input is a set of junctions to filter in tab format. This pipeline first creates a model from a high confidence set of likely genuine and likely false junctions. The model is then applied to the full set of junctions and output in tab and bed format.

The Portcullis filtering pipeline, shown in Fig. 10, as previously discussed, first creates an initial positive and negative training based on rule-based filtering. These sets allow us to train a new customized model for each dataset passed through Portcullis, allowing Portcullis to adapt to read length, read quality, sequencing depth, species, and mapping tool. Prior to training, the initial sets are then balanced using SMOTE [25], a synthetic oversampling technique, in order to partially compensate for biases that can be introduced to the model that favor the larger set. The balanced sets are used to train a random forest [26] using 100 trees, which we identified as providing a good balance between accuracy and runtime; see Supplementary Section 4 for justification. Finally, we apply the trained model to the full set of junctions in order to assign a probability score. Portcullis’ default behavior is for values >= 0.5 to be defined as genuine junctions and < 0.5 as invalid, although this threshold can be adjusted by the user to prioritize recall or precision.

## Availability of source code and requirements

- Project name: Portcullis
- Project home page: https://github.com/TGAC/portcullis
- Online documentation: http://portcullis.readthedocs.io/en/latest/
- Version at time of publication: V1.1.0
- Operating system(s): Unix based
- Programming language: C++11, Python V3.5+
- License: GNU GPL V3
- Supported package managers: Brew, Bioconda
- Research Resource Identification Initiative ID (RRID): SCR_016442

## Availability of supporting data

The datasets supporting the results of this article are available from the European Nucleotide Archive. For Arabidopsis we combined all samples from PRJEB7093 (http://www.ebi.ac.uk/ena/data/view/PRJEB7093). For Drosophila we combined all samples from SRA009364 (http://www.ebi.ac.uk/ena/data/view/SRA009364). For Homo Sapiens we combined all samples from PRJEB4208 (http://www.ebi.ac.uk/ena/data/view/PRJEB4208). Snapshots of the code and other supporting data are available in the *GigaScience* repository, GigaDB [27]. Additional results are available in the Supplementary Materials. We supply packages containing junctions present in the simulated datasets (derived from the previously mentioned datasets), along with the junctions extracted directly from BAM files and after filtering. These are available from figshare (https://figshare.com/projects/Portcullis/30779).

### Abbreviations

AS: alternative splicing; BAM: binary alignment map; DTA: Downstream Transcript Assembly; MaxMMES: maximum of the minimal match on either side of the splice junction; NGS: next-generation sequencing; RNA-seq: RNA sequencing; SAM: Sequence Alignment Map; SJ: splice junction .

### Competing Interests

The authors declare that they have no competing interests.

### Funding

This work was strategically funded by the Biotechnology and Biological Sciences Research Council (BBSRC), Core Strategic Programme (grant BB/CSP1720/1) at the Earlham Institute and by a strategic LOLA award (BB/J003743/1). NGS and library construction was delivered via the BBSRC National Capability in Genomics (BB/CCG1720/1) at Earlham Institute by members of the Genomics Pipelines Group.

### Author Contributions

The lead author is D.M. and the corresponding author is D.S. We describe contributions for all authors using the CRediT taxonomy. The order of authors for each task represents their relative contribution. Conceptualization: D.S. and D.M.; methodology: D.M. and D.S.; software: D.M. and L.V.; validation: D.M., L.V., G.K., and D.S.; writing the original draft: D.M.; writing–review and editing: D.M., D.S., L.V., and G.K.; visualization: D.M. and L.V.; supervision: D.S.

## Supplementary Material

Response-to-Reviewer-Comments_Revision-1.pdf

Response-to-Reviewer-Comments_Revision-2.pdf

Response_to_Reviewer-Comments_Original_Submission.pdf

Reviewer-1-Report-(Original-Submission) -- Pär Engström 5/10/2018 Reviewed

Reviewer-1-Report-Revision-1 -- Ricardo Ramirez-Gonzalez 1/10/2018 Reviewed

Reviewer-2-Report-(Original-Submission) -- Shihao Shen 17/9/2018 Reviewed

Supplement File

## Footnotes

1 A full set of false junctions would include every combination of start and stop sites in the genome that are not genuine junctions.

2 While this assumption is true for our selection of mappers, it may not always be true as different mappers (or mapper versions) may have different characteristics.
