# Supplementary material for: Efficient and accurate detection of splice junctions from RNA-seq with Portcullis
Source: Gigascience. 2018 Dec 12;7(12):giy131. doi: 10.1093/gigascience/giy131 (PMC6302956; doi:10.1093/gigascience/giy131)
Supplement: Supplement File [file giy131_supplement.pdf]

# Portcullis - Supplementary Information

Daniel Mapleson, Luca Venturini, Gemy Kaithakottil
and David Swarbreck.

August 11, 2018

## 1 Datasets

### 1.1 Simulated Read Generation

To generate simulated reads we constructed a pipeline utilising SPANKI (Sturgill et al., 2013) in order to incorporate realistic sequencing error profiles and expression levels. The steps in this pipeline are as follows:

1. Acquire genome file and reference annotation in GTF format for the species in question.
2. Use trimgalore http://www.bioinformatics.babraham.ac.uk/projects/trim_galore to remove any adaptors from the original dataset.
3. Index genome using Bowtie (Langmead et al., 2009) and HISAT (Kim et al., 2015)
4. Align dataset with Bowtie and HISAT
5. Create error model using SPANKI based on Bowtie alignments
6. Sort and index HISAT data
7. Assemble HISAT alignments with cufflinks
8. Extract expression levels from cufflinks and convert to SPANKI format
9. Create simulated reads and alignments using SPANKI based on annotation and cufflinks (Trapnell et al., 2010) expression levels
10. Use Portcullis to extract junctions from perfect SPANKI alignments.

The pipeline is complicated by the need to generate multiple datasets per species based on variable read lengths and expression levels. In order to retain a valid error model we can only use read lengths that are the same length or shorter than the original dataset. To vary expression levels we can take fractions or multiples of the cufflinks expression levels and run the read generation step multiple times. In addition, in order to speed the generation of simulated data we first chunk the expression levels, run the read generation tools and then merge the resulting fastqs and BAM files after.

### 1.2 Simulated data

To compare performance between junction filtering tools we created several simulated RNA-Seq datasets based on three known model organisms (Human, Drosophila and Arabidopsis), with error and expression

profiles derived from real datasets using SPANKIsim (Sturgill et al., 2013). This produces reads derived from a known region in the reference transcriptome, along with the perfect alignments of those reads. From the alignments it is possible to unambiguously derive the true set of junctions for the given dataset, providing a platform from which RNA-Seq mappers and SJ filtering tools can be benchmarked. The complete pipeline used to generate the simulated reads is described in section 1.1. Basic statistics for the datasets are described in table 1.

Table 1: Properties simulated datasets for each species

| Properties of simulated dataset | Arabidopsis | Drosophila | Human |
|---|---|---|---|
| Reference annotation | TAIR10 | DMEL78 | HG38 |
| Original accession | PRJEB7093 | SRA009364 | PRJEB4208 |
| Millions of reads (in original) | 93 | 47 | 97 |
| Mean quality (in original) | 37 | 37 | 39 |
| Depth fractions | 10%,50%,100% | 10%,50%,100% | 10%,50%,100%,200% |
| Millions of paired reads | 9,47,93 | 5,24,47 | 7,38,76,152 |
| Read lengths @ 100% depth (bp) | 76,101 | 76 | 76,101,151,201 |
| # splice junctions @ max readlen and 100% depth | 90,190 | 29,275 | 139,403 |
| % of SJ's in ref | 71% | 51% | 42% |
| # transcripts @ max readlen and 100% depth | 19723 | 9376 | 19853 |
| % of transcripts in ref | 47% | 32% | 12% |

# 2 Performance Evaluation Metrics

For most of the experiments described in this paper, our truth set consists of a subset of genuine junctions taken from a reference transcriptome. However, it is impractical to derive a comprehensive set of false junctions (true negatives $TNs$) (every combination of start and stop sites in the genome that are not genuine junctions). We therefore use the performance metrics recall, precision and $F_1$ measure (Fawcett, 2006)). Note the equations for these metrics do not require $TNs$ to be provided.

$$Recall = \frac{TP}{TP + FN} \tag{1}$$

$$Precision = \frac{TP}{TP + FP} \tag{2}$$

$$F_1 = \frac{2TP}{2TP + FP + FN} \tag{3}$$

Recall represents the fraction of true junctions retrieved, otherwise known as sensitivity or the true positive rate. Precision represents how often when the system makes a call it is correct, otherwise known as the positive predictive value. $F_1$ is the harmonic mean of recall and precision and a convenient proxy for the overall accuracy of the system.

# 3 Rule-based filtering of splice junctions

A simple strategy to filtering false positive junctions is to apply cut-off values to selected features. Figure 1 shows various rules individually applied to the TopHat2 alignments (black dot in figure) of our simulated Human 101bp dataset. Limiting junctions to those with an entropy greater than 2 as suggested

in SPANKI (Sturgill et al., 2013) does improve precision but can lead to an unacceptable loss of recall. Using a MaxMMES cutoff of 8 on the other hand will almost always provide better precision than the input with very little loss in recall. TopHat2's own filtered junction file in yellow. As a comparison we also show Portcullis' self-trained machine learning approach in red.

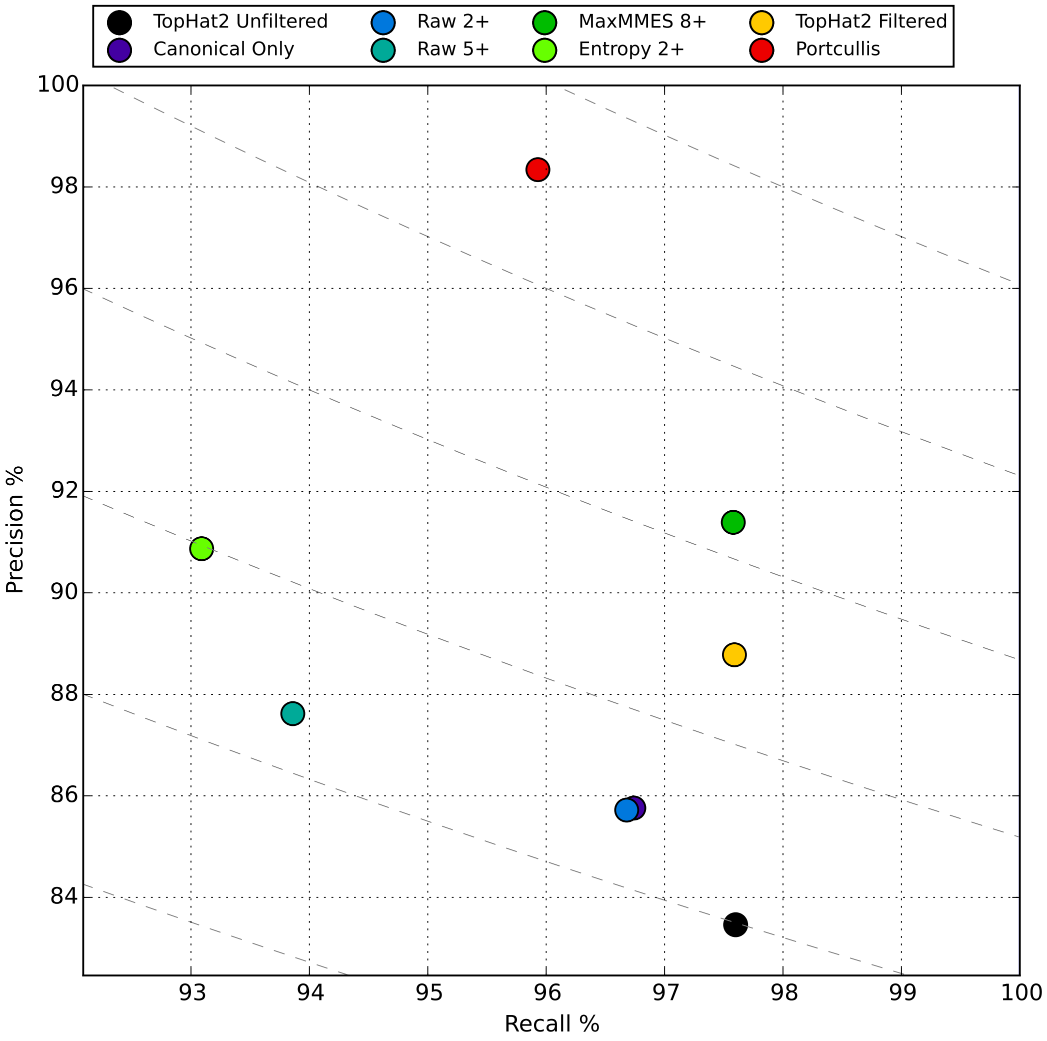


Figure 1: Effect on precision and recall of applying rules to junction metrics on TopHat2 alignments of our simulated Human 101bp dataset.

It is difficult to set cut-off values appropriate for all species and datasets. For example, setting a limit on intron size may be appropriate for one species but not another. Similarly, relying on the Shannon entropy score of the junction may produce good results on a deep sequenced dataset but poor results on a shallow one.

Although generally not recommended in most cases, Portcullis can interpret a user-define configuration that describes individual metrics selected for filtering, along with cut-off values. The user can chain rules together with 'and', 'or' and 'not' operations in order to come up with their own filtering strategy.

## 3.1 Using rule-based filtering to create training sets

Although, it is difficult to compete with the most accurate methods using rule-based approaches it is possible to select high-precision subsets using rules, which then can be used to train a machine learning approach. Portcullis uses a combination of rules to do exactly this. For our simulated human 101bp

dataset, our rules for extracting the positive set containing 111,217 genuine junctions achieves a precision of 99.94% with recall of 80.05%. Rules for collecting the negative set contains 13,870 false junctions compared to only 63 true ones. To achieve good results with using machine learning, it is important to capture representative subsets of both true and false junctions, while keeping near perfect precision. It is also important the rules work over a range of datasets and species. In Portcullis we try to accomplish this using multiple rules. Portcullis version 1.1.2 uses the following set of rules for creating the positive set:

- Layer 1 ( Reliable alignments >= 1 & MaxMMES >= 8 & Entropy > 1 & Hamming 5' >= 4 & Hamming 3' >= 4 & Mean mismatches <= 1 & Uniquely spliced alignments >= 1 & Reliable to raw alignment ratio >= 0.25 )
- Layer 2 (( Reliable alignments >= 5 & MaxMMES >= 20 ) | ( Reliable alignments >= 3 & MaxMMES > 12 & Hamming 5' >= 7 & Hamming 3' >= 7 & Mean mismatches < 0.33 ) | ( Hamming 5' >= 9 & Hamming 3' >= 9 & Mean mismatches == 0 ))
- Layer 3 ((( Is canonical ) | ( Is semi-canonical & reliable to raw ratio >= 0.75 & Hamming 5' >= 6 & Hamming 3' >= 6 ) | ( Is non-canonical & reliable to raw ratio >= 0.75 & Hamming 5' >= 7 & Hamming 3' >= 7 & Mean mismatches < 0.1 & Entropy > 1.5 )) | ( Is primary junction ))

The result of each layer in the positive set is treated as input to the second, effectively acting as an ”and” operation. For the negative set, each layer is treated as an ”or” operation. The negative rules are as follows:

- Layer 1 ( MaxMMES < 15 & Uniquely spliced reads <= 1 & Reliable to raw ratio == 0 )
- Layer 2 ( Uniquely spliced reads <= 1 & MaxMMES < 15 & ( Is NOT canonical | Mean mismatches >= 1 ) )
- Layer 3 ( Is NOT canonical & Is Potential False Positive )
- Layer 4 ( MaxMMES < 15 & Reliable to raw ratio < 0.3 )
- Layer 5 ( Reliable alignments == 0 & Entropy == 0 & Is NOT primary junction & Is suspicious )
- Layer 6 - currently unused
- Layer 7 ( Reliable 2 raw ratio == 0 & Hamming 5' <= 3 & Hamming 3' <= 3 )

# 4 Deciding on a classifier

For classifying junctions we considered two widely used machine learning methods: logistic regression with L1 regularisation (Ng, 2004) and random forests (Breiman, 2001). Deep learning frameworks we not considered at this time primarily due to computational cost, portability concerns and maturity at time of development, although we plan to look into these in the future.

Across all our simulated datasets and aligners we found the mean $F_1$ measure to be similar for both methods: Logistic regression = 97.50% and random forests = 97.44%. Despite a slightly lower score, we chose a random forest as the learning method to build into Portcullis because they are suitable for handling categorical variables, which means we can more easily extend our learning model with new categorical features in the future. An additional benefit of Random Forests is that they do not have as many hyperparameters that require optimisation. The actual random forest implementation used inside Portcullis is derived from an R package called ranger (Wright and Ziegler, 2015).

A Random Forest is a method that uses random sampling and random feature selection of the input feature vectors to produce an ensemble of decision trees. In our context, we take the mean of probability

scores produced from all trees in the forest to provide a way to differentiate between a genuine and invalid SJ. A score close to 0 indicates an invalid junction and close to 1 a genuine SJ.

One parameter that requires optimising in a Random Forest is the number of trees in the forest. To determine the optimal number we trained and executed a random forest classifier across our various simulated datasets with inputs from TopHat2, GSNAP, STAR and HISAT2. In this experiment we look at classification performance based off off each input aligners junction set. This enables us to definitely count the number of True Negative's in each dataset and in turn allows us to use Matthew's Correlation Coefficient (Powers, 2011), as defined in equation (4). This is a better measure of the classifiers predictive performance when class sizes are very different, which is often the case for us.

$$Matthew's\, Correlation\, Coefficient\, (MCC) = \frac{TP \cdot TN - FP \cdot FN}{\sqrt{(TP+FP)(TP+FN)(TN+FP)(TN+FN)}} \quad (4)$$

The mean MCC after 10-fold cross validation across each mapper is shown in figure 2. The MCC appears to plateau at 100 trees across all species so this is set as the default value when running Portcullis.

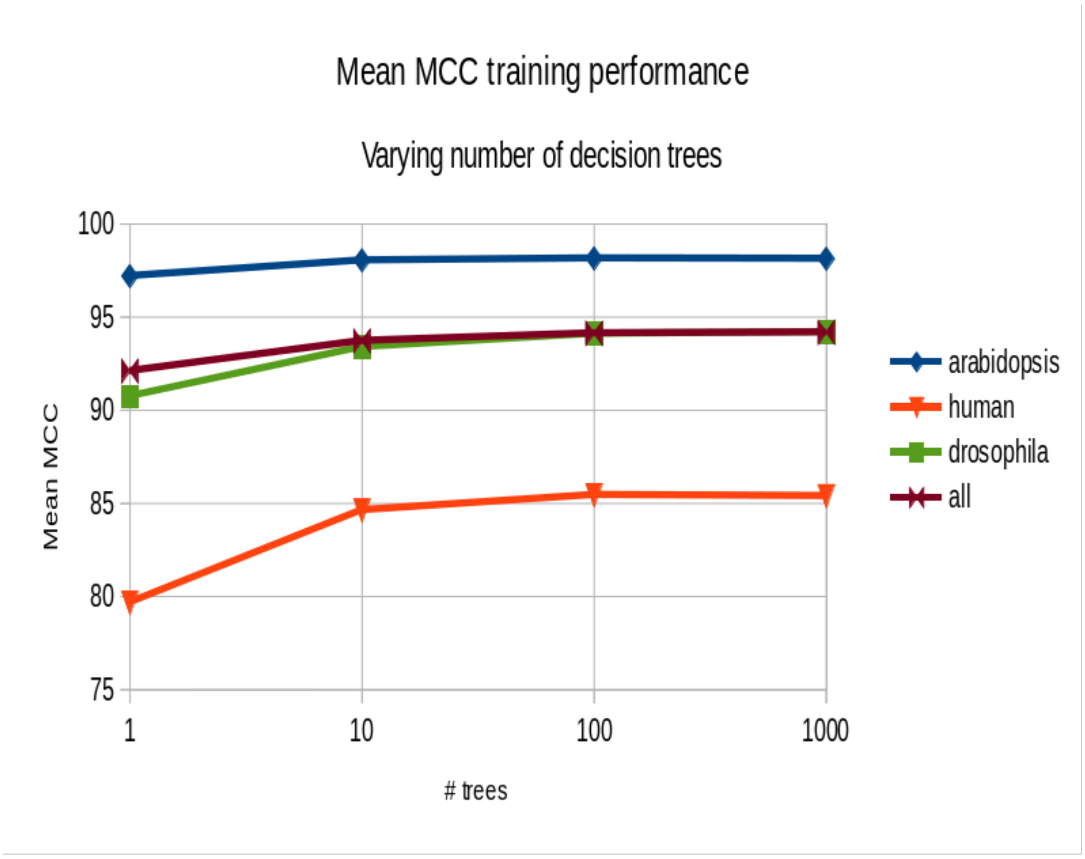


Figure 2: Matthew's Correlation Coefficient with respect to the number of trees in the random forest run datasets from for each species separately as well as with a combined dataset. 10-fold cross validation was used.

# 5 Junction Detection Performance

## 5.1 Effect of dataset on SJ accuracy

Figure 3 shows the effect of varying dataset size and read lengths across multiple mapping methods on variations of our simulated human dataset. Although each mappers performance varies the same trends occur in each case: increased depth produces more false positive junctions, and increased read length enables the methods to recall more genuine junctions and at the same time generally improve precision. The only data point where this trend is not supported is with HISAT2 201bp reads, where precision is slightly less than at 151bp, indicating that HISAT2 may have problems with longer reads that span multiple junctions.

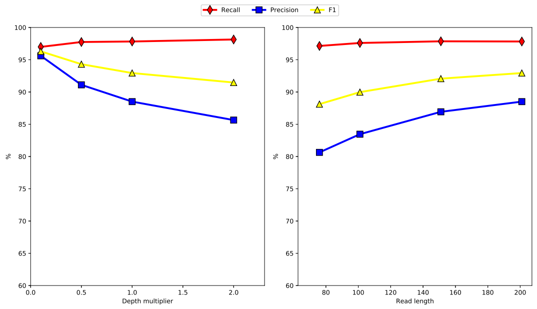


(a) TopHat v2.1.0

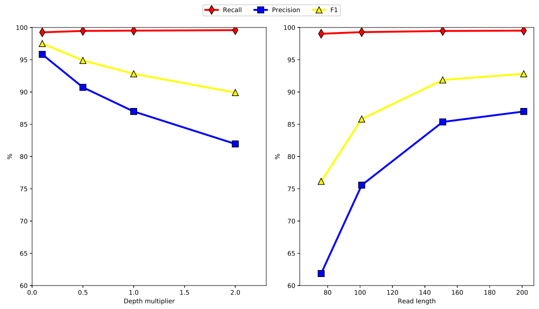


(b) GSNAP v20180530

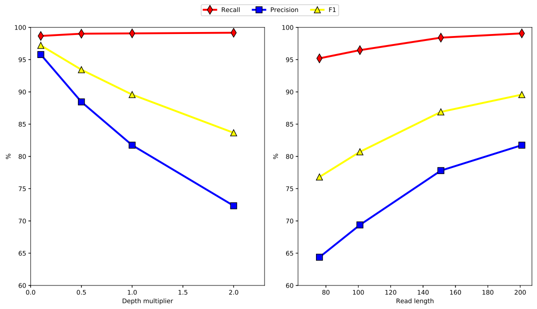


(c) STAR v2.6.0a

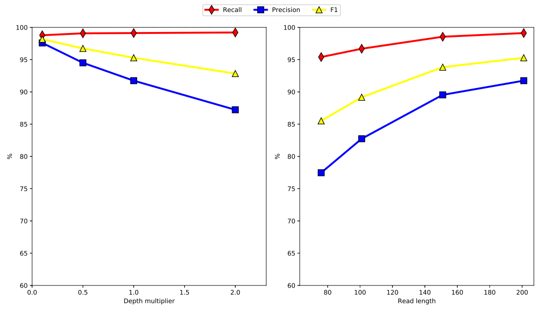


(d) STAR (2Pass) v2.6.0a

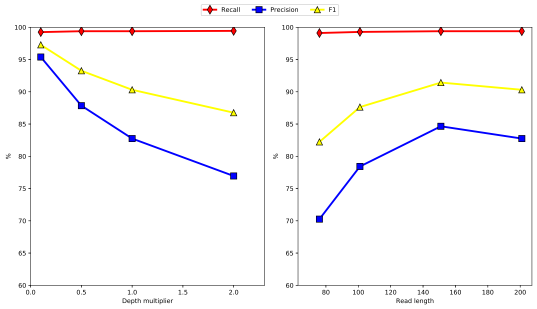


(e) HISAT v2.1.0

Figure 3: In this plot we show the effect on recall and precision of junction calling for multiple alignment methods over variations of our simulated human dataset. Left-side of each sub-plot shows the effect of varying dataset size, with all datasets containing 201bp reads. 1.0X depth multiplier represents a dataset of ∼76 million read pairs. The right-side of each sub-plot shows the effect of varying read length with all datasets containing ∼76 million read pairs.

## 5.2 Agreement between mappers

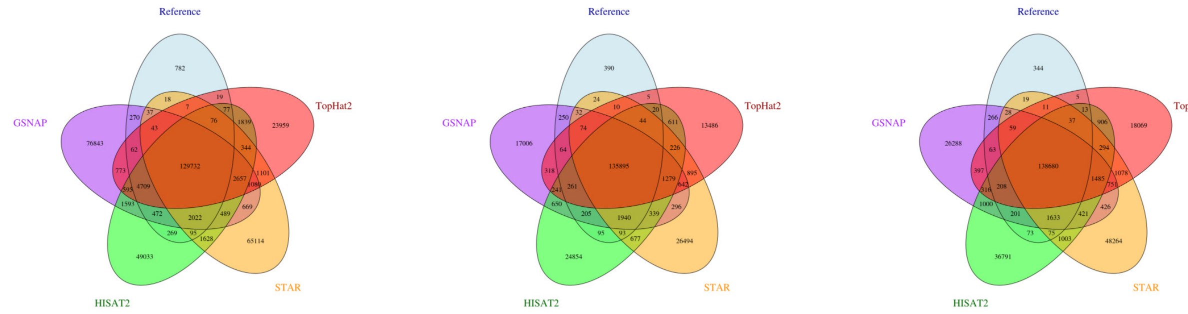


(a) ∼30Gbp of 76bp reads (b) ∼30Gbp of 201bp reads (c) ∼60Gbp of 201bp reads

Figure 4: Five way Venn diagrams showing levels of agreement between mapping tools and the human junction truth set. (a) shows agreement with 76bp simulated reads, (b) shows agreement with 201bp simulated reads with the same dataset size as (a): ∼30Gbp. (c) Shows 201bp reads at twice the depth of (b).

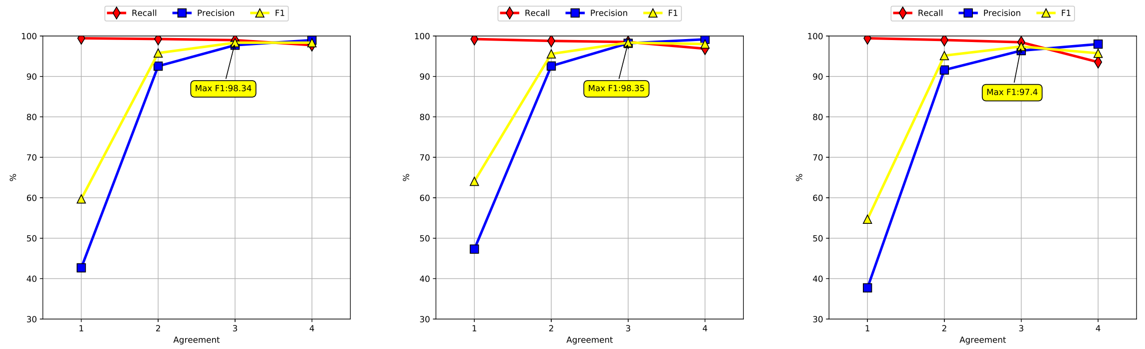


(a) Arabidopsis 76bp (b) Drosphila 76bp (c) Human 76bp

Figure 5: Junction set results after requiring consensus between a varying number of mappers: TopHat2, GSNAP, STAR and HISAT2. Agreement level 1 is the union of junctions found in all mappers. Agreement level 4 is the intersection of junctions found in all mappers. All input datasets were from simulated 76bp paired reads.

## 5.3 Accuracy of splice site prediction tools

Figures 6 to 8 show how $F_1$ scores vary between methods when varying sequencing depth and read length and species. This analysis highlights some notable characteristics of the methods we tested. First, SOAPsplice performs well with read lengths $<= 101bp$ but performance degrades significantly with longer reads; likely due to a failure of handling reads containing multiple splice junctions. Second, MapSplice, FineSplice and Portcullis show consistent and reliable behaviour across datasets. Third, as expected the RNA-Seq mappers fall far behind the more computationally expensive tools, particular at high depth and low read length. Fourth, TrueSight is difficult to use due to long runtimes and high memory usage (see section 5.4 for more information), meaning that we could not collect results for this method from many datasets. Fifth, TopHat2's own rule-based filtering approach provides limited improvement to accuracy. Finally, Portcullis improves results over input mappers and is either competitive or has the highest $F_1$ in all cases.

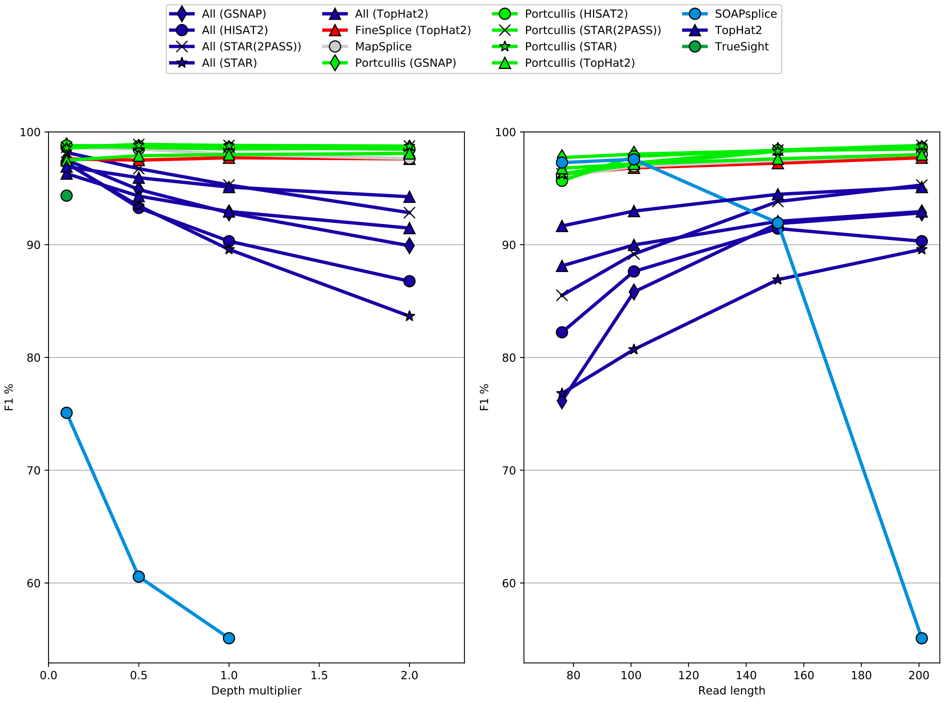


(a) Full

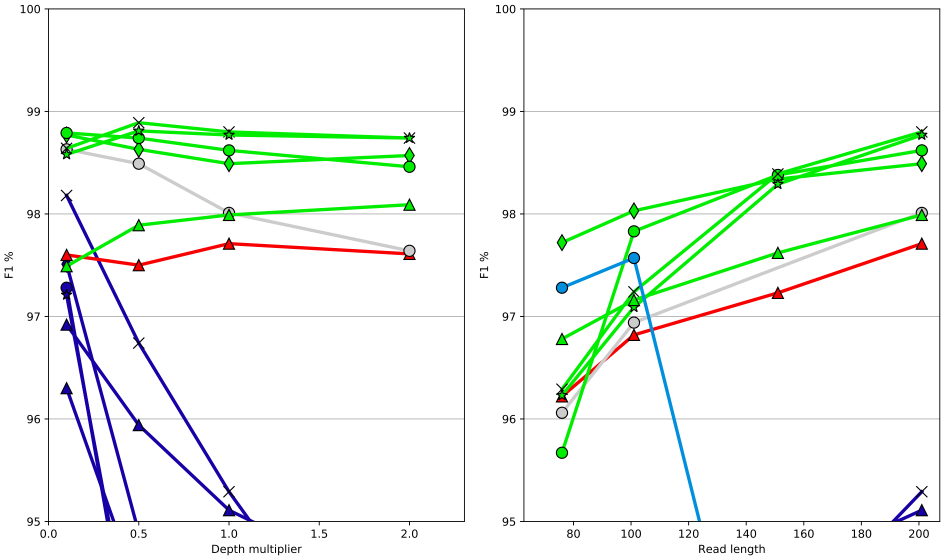


(b) Zoomed

Figure 6: $F_1$ scores across all Human simulated datasets and all methods. Left-hand side shows effect of varying depth, right hand side shows varying read length. Depth variations all used 201bp reads. Read length variations used a 1X expression multiplier of a dataset containing ∼76 million reads, so each read length variation contains the same number of bases in the input. Not all TrueSight runs completed, as we automatically killed jobs after one week of processing. (a) shows the full set of results and (b) shows $F_1$ scores over 94% to show the characteristics of the best performing methods.

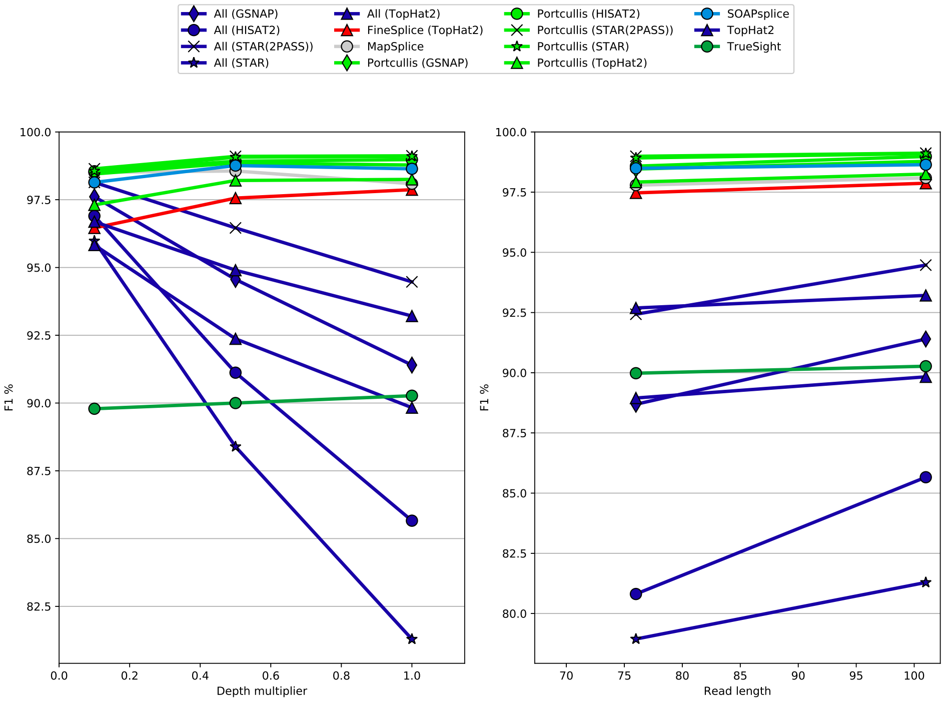


(a) Full

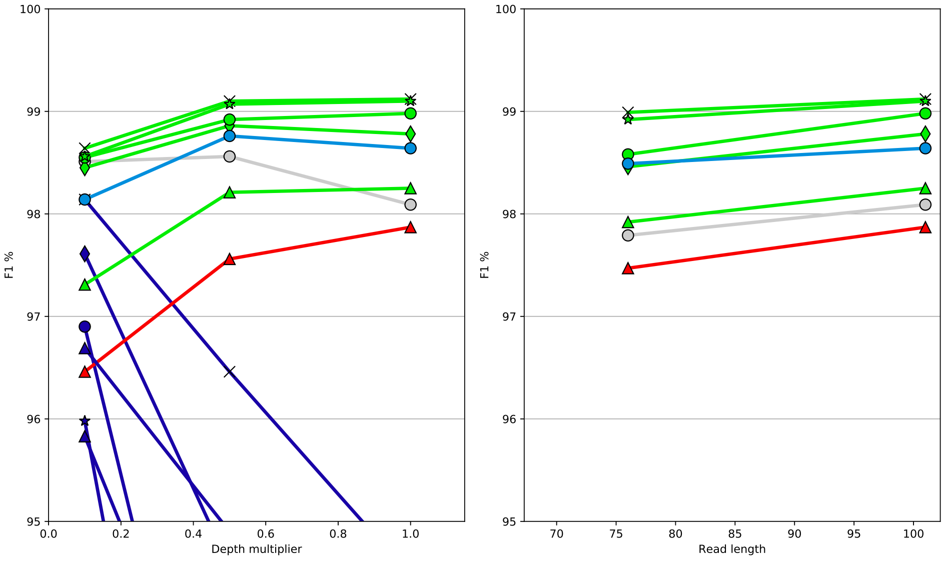


(b) Zoomed

Figure 7: $F_1$ scores across all Arabidopsis simulated datasets and all methods. Left-hand side shows effect of varying depth, right hand side shows varying read length. Depth variations all used 101bp reads. Read length variations used a 1X expression multiplier of a dataset containing ~93 million reads, so each read length variation contains the same number of bases in the input. (a) shows the full set of results and (b) shows $F_1$ scores over 95% to show the characteristics of the best performing methods.

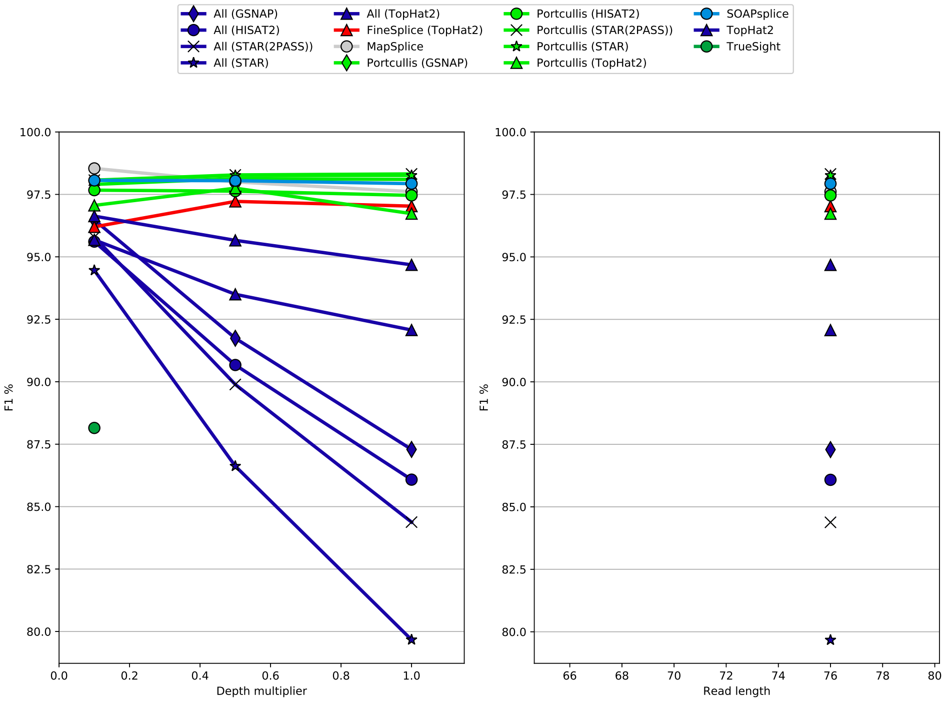


(a) Full

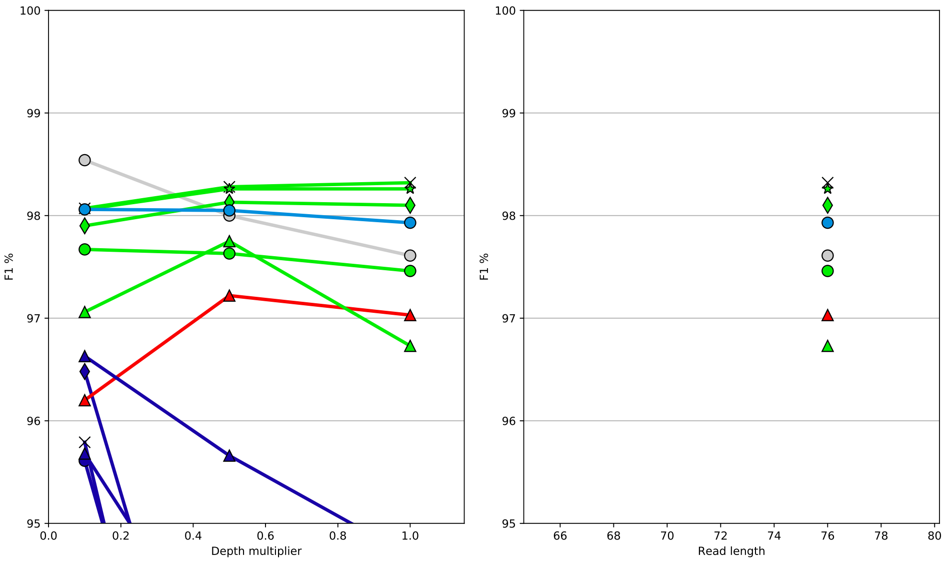


(b) Zoomed

Figure 8: $F_1$ scores across all Drosophila simulated datasets and all methods. Left-hand side shows effect of varying depth, right hand side shows varying read length. Depth variations all used 76bp reads. Read length variations used a 1X expression multiplier of a dataset containing ∼47 million reads, so each read length variation contains the same number of bases in the input. (a) shows the full set of results and (b) shows $F_1$ scores over 95% to show the characteristics of the best performing methods.

## 5.4 Runtimes of splice site prediction tools

In general we see Portcullis as being relatively frugal in terms of memory usage. However, we note that its memory usage does increase in deep datasets, becoming more obvious when using more frugal aligners such as HISAT2. This can be seen in figure 9 at 2X depth and reflects the way Portcullis processes the BAM files, where each thread processes reads aligning to different reference sequences. This requires all reads supporting a given junction to be in memory at a given time, for each running thread. All examples here were run with 8 threads, so a simple way to reduce memory usage is to reduce the number of threads used, although this will have an impact on runtime.

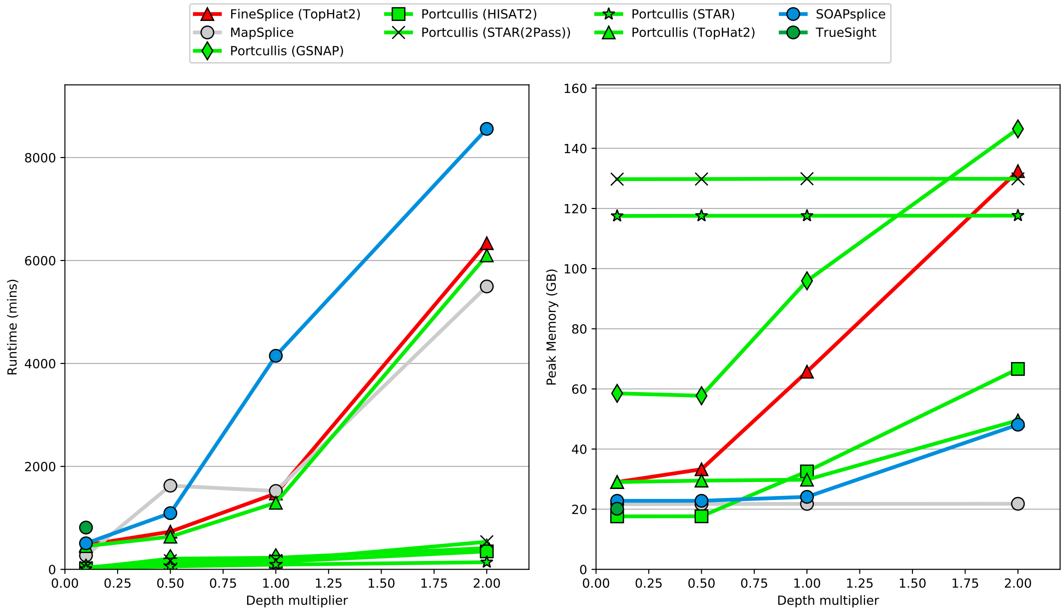


Figure 9: Runtimes and max memory usage of Portcullis when varying read length on our Human dataset containing ∼76 million paired reads.

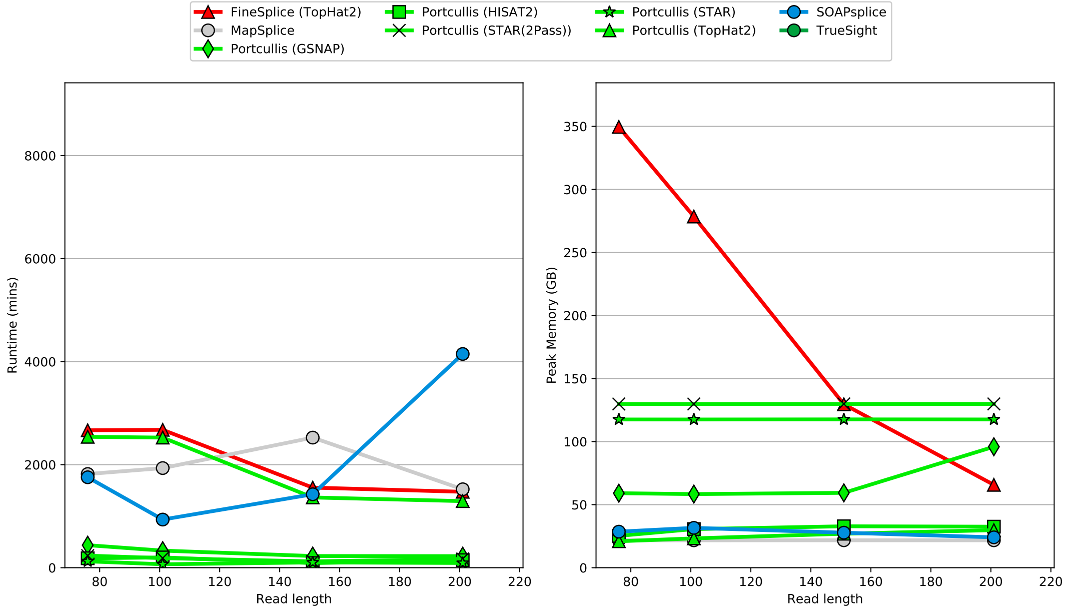


Figure 10: Runtimes and max memory usage of Portcullis when varying read length on our Human dataset containing ∼76 million paired reads.

To analyse this runtime / memory trade off in more detail, we plotted runtime and memory usage as the

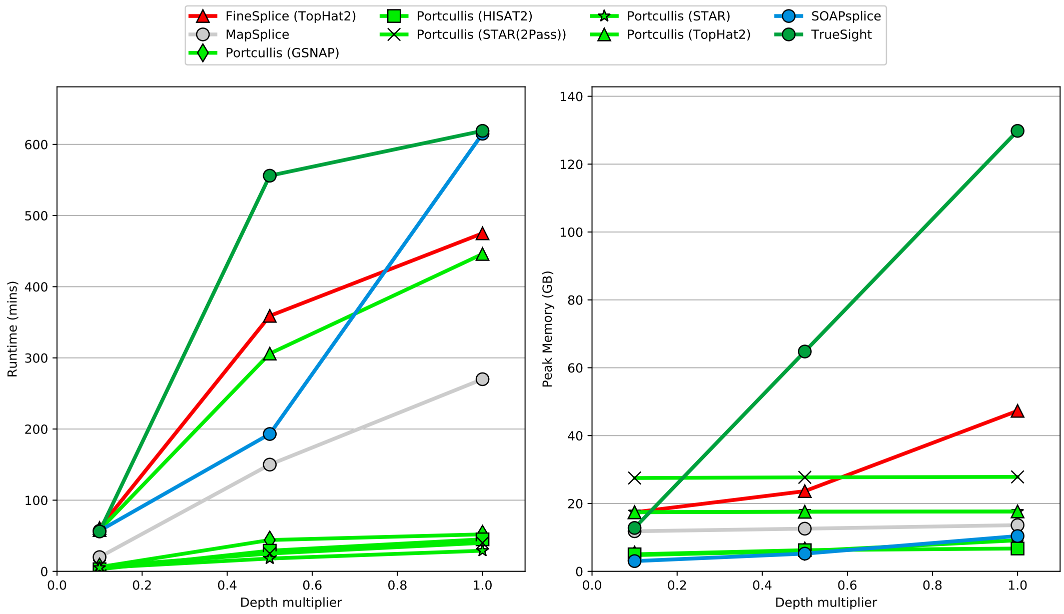


Figure 11: Runtimes and max memory usage of Portcullis when varying depth on our 101bp paired-end Arabidopsis dataset.

number of threads is varied on our human dataset with ∼76 million 201bp paired reads (figure 12). All axis are logscale to show whether memory and runtime scale linearly, indeed memory appears to scale linearly with thread count, approximately doubling when jumping from using 1 to 16 threads. In terms of runtime, Portcullis scales well up to 4 threads after which point diminishing returns are encountered, particularly with deeper datasets.

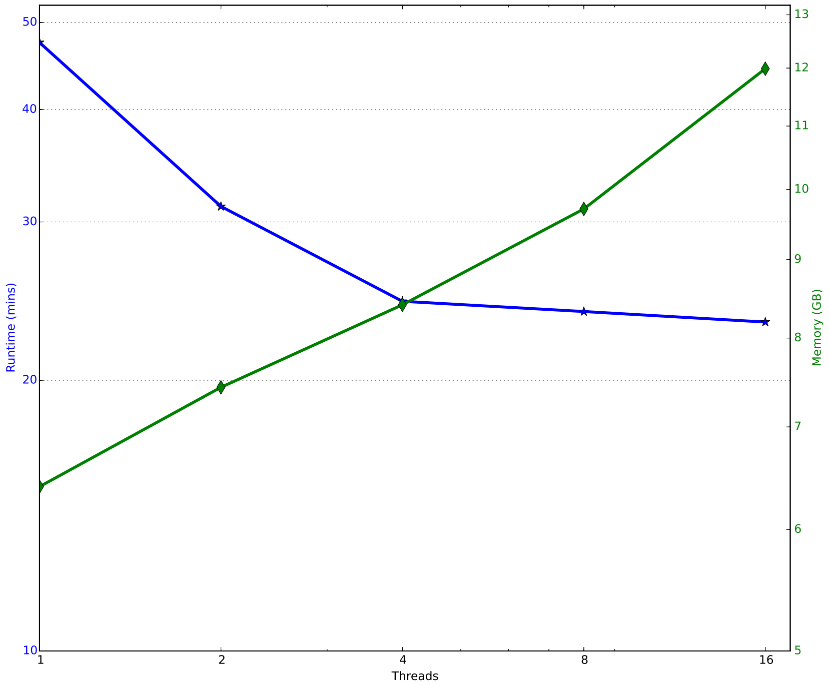


Figure 12: Portcullis' runtime and max memory usage when varying thread count on our human dataset containing 80 million 201bp paired reads.

### 5.5 Analysis of real data

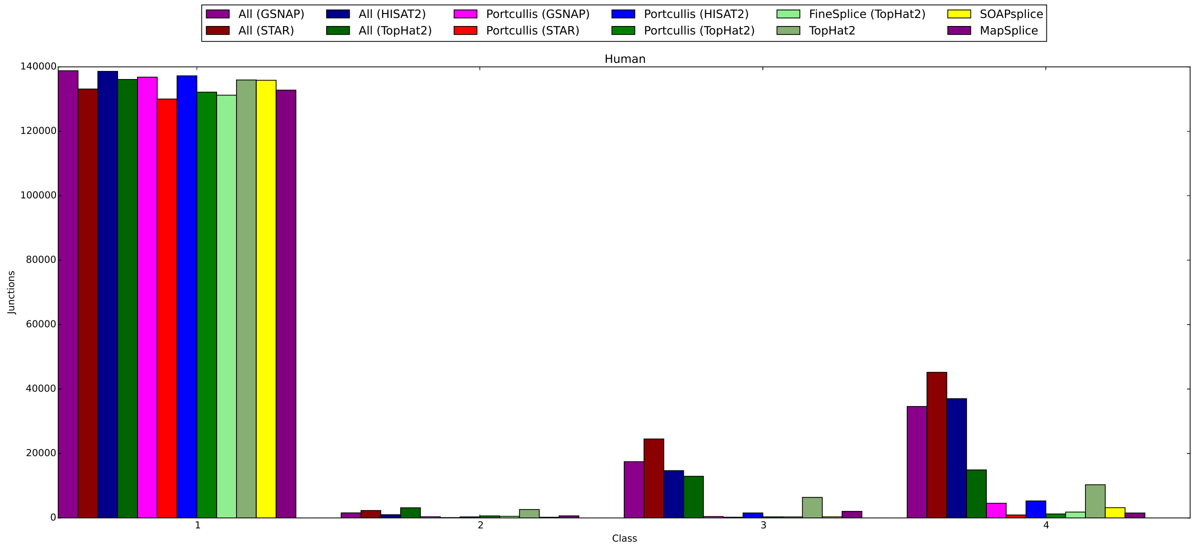


Figure 13: This plot shows a class breakdown of junctions found using simulated 76bp reads from the human dataset. Correct junctions belong exclusively to class 1, where as classes 2,3 and 4 show false positives.

## 6 Junctools

Portcullis comes bundled with a supplementary toolkit called Junctools, which provides the user with a number of features for manipulating and analysing junction files in many commonly used formats.

Convert - Junctools can convert between commonly used junction files, such as BED and GFF, or between these and input to guide several popular RNA-Seq mappers so they can be easily driven in two-pass mode without requiring the user to do their own scripting.

Compare - Junctools can compare junction files against each other, which can be useful to see how many junctions from Portcullis are present in a given reference.

Set operations - In addition, Junctools supports set operations between junction files, which can be useful for merging results between several Portcullis runs, or to separate junctions not found in a reference annotation for example.

GTF - Finally Junctools offers the user the ability to filter out or markup transcripts in GTF files, that do not contain junctions found in a separate junctions file. This allows the user to filter out transcripts that do not have junctions that are supported by Portcullis for example.

## 7 List of Software

This section outlines the software and command lines used for experiments in this paper. For reasons of brevity we have used variables and kept listed the static parts of the command lines. Variables we used to modify species, datasets and runtime characteristics of the tool. The variables related to species are were MIN_INTRON and MAX_INTRON are are listed in Table 2. THREADS, was generally set at 8, except when varying thread count as shown in Figure 12. The remaining variables, vary according to the dataset used.

Table 2: Species associated variables

| Variable | Arabidopsis | Drosophila | Human |
|---|---|---|---|
| MIN_INTRON | 50 | 50 | 50 |
| MAX_INTRON | 50,000 | 50,000 | 500,000 |

The following RNAseq aligners were used for experiments in this:

- Tophat v2.1.0
  - Indexing: bowtie2-build REF ALIGN_DIR/tophat/index/NAME
  - Running: tophat2 –output-dir=OUTDIR –num-threads=THREADS –min-intron-length=MIN_INTRON –max-intron-length=MAX_INTRON –microexon-search –library-type=STRAND INDEXDIR R1 R2
- GSnap v20180530
  - Indexing: gmap_build –dir=ALIGN_DIR/gsnap/index –db=NAME INPUT
  - Running: gsnap –gunzip –dir=ALIGN_DIR/gsnap/index –db=NAME –novelsplicing=1 –localsplicedist=MAX_INTRON –nthreads=THREADS –format=sam –npaths=20 R1 R2
- STAR v2.6.0a
  - Indexing: STAR –runThreadN THREADS –runMode genomeGenerate –genomeDir INDEXDIR –genomeFastaFiles INPUT
  - Running: STAR –readFilesCommand zcat –runThreadN THREADS –runMode alignReads –genomeDir INDEXDIR –readFilesIn R1 R2 –outSAMtype BAM Unsorted –outSAMstrandField intronMotif –alignIntronMin MIN_INTRON –alignIntronMax MAX_INTRON –alignMatesGapMax 20000 –outFileNamePrefix OUTDIR/
  - Running (2pass): STAR –readFilesCommand zcat –runThreadN THREADS –runMode alignReads –genomeDir INDEXDIR –readFilesIn R1 R2 –outSAMtype BAM Unsorted –twopassMode=Basic –outSAMstrandField intronMotif –alignIntronMin MIN_INTRON –alignIntronMax MAX_INTRON –alignMatesGapMax 20000 –outFileNamePrefix OUTDIR/
- HISAT v2.1.0
  - Indexing: hisat2-build INPUT ALIGN_DIR/hisat/index/NAME
  - Running: hisat2 -p THREADS –min-intronlen=MIN_INTRON –max-intronlen=MAX_INTRON STRAND -x INDEXDIR -1 R1 -2 R2

For splice junction aligners:

- Finesplice v0.2.2: FineSplice.py -i BAM -l READLEN
- Truesight v0.06: truesight_pair.pl -i MIN_INTRON -I MAX_INTRON -v 1 -r INDEX -p THREADS -o . -f R1 R2
- Soapsplice v1.10: soapsplice -d INDEX -1 R1 -2 R2 -I 500 -o OUTDIR/ss-READS -p THREADS -t MAX_INTRON -c 0 -f 2 -L MAX_INTRON -l MIN_INTRON
- Mapsplice v2.2.1: mapsplice.py -c REFDIR -1 R1 -2 R2 -o OUTDIR/ -p THREADS –bam -i MIN_INTRON -I MAX_INTRON

Portcullis v1.1.2 was run with each stage executed independently for maximum flexibility:

- Prep stage: portcullis prep -o OUTDIR -t THREADS REF BAM
- Analysis stage: portcullis junc -o OUTDIR/PREFIX –orientation=FR –strandedness=STRAND -t THREADS PREPDIR
- Filter stage: portcullis filter -t THREADS -o OUTDIR/PREFIX PREPDIR TAB

Transcript assemblers:

- Cufflinks v2.2.1: cufflinks –output-dir=OUTDIR –num-threads=THREADS –library-type=STRANDEDNESS –min-intron-length=MIN_INTRON –max-intron-length=MAX_INTRON -F ISO_FRAC –no-update-check BAM
- Stringtie v1.3.0: stringtie BAM -l NAME -f ISO_FRAC -m 200 -o GTF -p THREADS

# References


Breiman, L. (2001). Random forests. Machine learning, 45(1), 5–32.

Fawcett, T. (2006). An introduction to ROC analysis. Pattern Recognition Letters, 27(8), 861–874.

Kim, D., Langmead, B. and Salzberg, S.L. (2015). HISAT: a fast spliced aligner with low memory requirements. Nature Methods, 12(4), 357–360.

Langmead, B. et al (2009). Ultrafast and memory-efficient alignment of short DNA sequences to the human genome. Genome Biology, 10(3), R25.

Ng, A.Y. (2004). Feature selection, L1 vs. L2 regularization, and rotational invariance. In Proceedings of the twenty-first international conference on Machine learning, page 78. ACM.

Powers, D.M.W. (2011). Evaluation: from precision, recall and f-measure to roc, informedness, markedness and correlation. International Journal of Machine Learning Technology, 2(1), 37–63.

Sturgill, D. et al (2013). Design of RNA splicing analysis null models for post hoc filtering of Drosophila head RNA-Seq data with the splicing analysis kit (Spanki). BMC Bioinformatics, 14, 320.

Trapnell, C. et al (2010). Transcript assembly and quantification by RNA-Seq reveals unannotated transcripts and isoform switching during cell differentiation. Nature Biotechnology, 28(5), 511–515.

Wright, M.N. and Ziegler, A. (2015). Ranger: A Fast Implementation of Random Forests for High Dimensional Data in C++ and R. arXiv.
