# Supplementary material for: Efficient and accurate detection of splice junctions from RNA-seq with Portcullis
Source: Gigascience. 2018 Dec 12;7(12):giy131. doi: 10.1093/gigascience/giy131 (PMC6302956; doi:10.1093/gigascience/giy131)
Supplement: Response-to-Reviewer-Comments_Revision-1.pdf [file giy131_response-to-reviewer-comments_revision-1.pdf]

Author's Response To Reviewer Comments

Close

We thank the reviewer for their comments and have addressed the remaining minor point (see below)

Reviewer reports:
Reviewer #1: I only have one remaining comment, relating to my previous comment 3. In figure 4, the use of the term "2PASS" in the labels is misleading, as its meaning in this figure differs between STAR and HISAT2. For STAR, it signifies the use of a built-in 2-pass mode. In contrast, for HISAT2 it signifies running HISAT2 a second time using input from portcullis. The arrows in the figure indicate this, but the terminology is still confusing. To fix this, I suggest changing the label "HISAT2(2PASS)" to "HISAT2 - Portcullis - HISAT2" and changing "HISAT2(2PASS) - Portcullis" to "HISAT2 - Portcullis - HISAT2 - Portcullis", or perhaps "(HISAT2 - Portcullis) x 2" for brevity. That would be consistent with the structure of the other labels in the figure (using dashes to signify that one program is run after another), and would be clearer for the reader. In addition, I suggest clarifying this aspect in the main text. For example, after the sentence ending in "…during the alignment process" on page 4, one could add a sentence "The effect of applying this strategy with HISAT2 is shown in Figure 4 (upper right corner), and it could be similarly applied with other mappers".

We have made the suggested changes to figure 4 and also include an additional sentence in the main manuscript as requested

"The effect of applying this strategy with HISAT2 is shown in Figure 4 (upper right corner), and it could be similarly applied with other mappers."

Close
