# Supplementary material for: Efficient and accurate detection of splice junctions from RNA-seq with Portcullis
Source: Gigascience. 2018 Dec 12;7(12):giy131. doi: 10.1093/gigascience/giy131 (PMC6302956; doi:10.1093/gigascience/giy131)
Supplement: Response-to-Reviewer-Comments_Revision-2.pdf [file giy131_response-to-reviewer-comments_revision-2.pdf]

Author's Response To Reviewer Comments

Close

We have made the edits indicated by your correspondence below

GIGA-D-18-00126R2
Efficient and accurate detection of splice junctions from RNA-Seq with Portcullis
Daniel Mapleson; Luca Venturini; Gemy Kaithakottil; David Swarbreck
GigaScience

Dear Dr Swarbreck,

I see our curation team have now released your dataset accompanying your manuscript

GIGA-D-18-00126
Efficient and accurate detection of splice junctions from RNA-Seq with Portcullis

I've just seen the DOI which needs to be added to your paper in the references list and also cited under "availability of supporting data".

As your manuscript is created in latex, I'm unable to add this information myself before sending to production. Can you please edit your paper and add the DOI and data sentence as per the PDF marked up attached?

Final revisions to be made are:
1. Add a final sentence under "Availabililty of supporting data" to read as "Snapshots of the code and other supporting data are available in the GigaScience repository, GigaDB [27]."

2. Add GigaDB DOI citation as reference 27:

27. Mapleson DL; Venturini L; Kaithakottil GG; Swarbreck D: Supporting data for "Efficient and accurate detection of splice junctions from RNA-Seq with Portcullis" GigaScience Database. 2018. http://dx.doi.org/10.5524/100519

The due date for submitting the revised version of your article is 19 Oct 2018.

Please upload your new version and associated files - then I will send it to Production.

Best wishes
Nicole

Close
