# Supplementary material for: Efficient and accurate detection of splice junctions from RNA-seq with Portcullis
Source: Gigascience. 2018 Dec 12;7(12):giy131. doi: 10.1093/gigascience/giy131 (PMC6302956; doi:10.1093/gigascience/giy131)
Supplement: Response_to_Reviewer-Comments_Original_Submission.pdf [file giy131_response_to_reviewer-comments_original_submission.pdf]

Author's Response To Reviewer Comments

Close

Dear Nicole Nogoy,

We have now revised our manuscript addressing the comments of yourself and the reviewers. We note that both reviewers indicated our tool would be useful to the community and thank them for their positive reviews, we have addressed the reviewers queries and provide a point by point response in reply. The main concern we have addressed by updating the referenced tools to more recent versions, updating figures and text as required. We provide a version of the manuscript with changes highlighted.

Editor comment 1

Portcullis has been registered on SciCrunch, and has been assigned RRID:SCR_016442. The "Availability of source code and requirements" section has been updated to include this RRID.

Reviewer reports:

Reviewer #1:

Comment 1. "Newer versions of the alignment programs should be used. The STAR, GSNAP and HISAT2 versions used are from 2016. For all three programs, there are later releases from 2017, and for STAR and GSNAP also from 2018. Even if the study might have been initiated in 2016, it should be straightforward to rerun the analyses with updated software versions, to confirm that the results are current. The TopHat version is also not the most recent, but that is less relevant since TopHat has been superseded by HISAT2."

We have rerun the aligners with their latest versions (as of June 2018) and updated all affected plots in the paper to reflect the new results. There was very little change in the results for STAR and HISAT2 being almost identical to the previous run. However, GSNAP results were actually worse with an F1 of 76.15% in our human 76bp dataset down from 83.07%. Similar trends were seen in other datasets.

Comment 2. "For this type of analysis, STAR should be run in 2-pass mode, since that makes it more accurate with regards to splice junctions. It would be good to check if GSNAP now has such a mode as well (last time I used GSNAP it didn't, but that was several years ago). HISAT2 uses a 2-pass strategy by default."

We have also run STAR in 2-pass mode and added it to the results. Performance of STAR in 2-pass mode, while improved, is still relatively mediocre relying on a very simple filtering strategy, before passing those junctions back in for a second pass. Results are shown clearly in Figure 4, and a similar trend follows in other datasets (see Supp fig 6-8). GSnap does not have an automatic 2-pass mode to our knowledge so we have not included additional results.

Comment 3. "Page 4: "Furthermore, we can improve F1 even more by running HISAT2 in a

two pass configuration, feeding in portcullis predicted junctions in the second pass." For fairness, it would be good to mention that this strategy isn't limited to HISAT2, but could be used with any mapper that accepts a list of known junctions as input (including STAR and GSNAP)."

We agree that most tools do have a manual 2-pass option where junctions can be provided as a guide. As noted we have not provided extensive analysis of the results from all tools after passing in portcullis filtered junctions, although we do show the effect of running HISAT2 in this way in Figure 4. In the manuscript we make the point that minor additional improvements in junction accuracy can be made this way, with the added benefit of producing cleaner alignments in the BAM file, albeit at the expense of runtime.

Comment 4. "Page 10: "To show that these additional features…". Which are the additional features referred to here? Please clarify. The preceding paragraphs don't seem to distinguish between additional and other features."

See our comment to point 5

Comment 5. "Page 10: "The left column (showing results without extracted features) has more overlap between genuine and invalid junctions than the middle column (with extracted features)." I don't think differences in overlap are apparent from the figure. Due to the many overlapping data points, the extent of separation between classes is difficult to assess visually. Could this be presented in some other way? Could the overlap (or separation) be quantified?"

To address these last two points,we have decided to remove this plot and the related text. It is well established in machine learning literature that careful feature engineering can improve classification performance and this point doesn't need to be made in the paper.

Comment 6. "Please specify the options used for each mapper in Methods or Supplement."

We have added a section to the supplementary which lists the main tools used within the paper, the versions used and the general command lines used to execute the tools as part of our pipeline. Where necessary we have left variables in the command lines for the sake of conciseness.

Comment 7. "If I were to review a revised version, I would appreciate if a larger font was used."

The style is defined by gigascience and altering font sizes across the entire stylesheet is quite an undertaking. However, we have created a version of the paper that highlights the differences between the previously submitted version and this version as a pre-made pdf. In that copy it was straightforward to disable the two column layout, so hopefully that should make it easier to review.

Reviewer #2:

Comment 1. "The authors introduced their methods for the accurate detection of splicing junction from RNA-seq data. The proposed method was based on the classification of

splicing junctions into multiple categories of different confidentialities. The categories were based on the novelty of the splicing junction (i.e. whether the junction is annotated in the gene transcript annotation file) and novelty of the splice site (i.e. whether the splice site is annotated).

The classification of splicing junctions is very useful to the field of RNA-seq analysis. It is expected that junctions with novel sites are less reliable than junctions with known sites. However, it would be too arbitrary to simply filter these junctions, especially in human subjects, since many novel events are associated with disease development."

The classification-based filtering system could be too stringent for some studies, although the classification itself is useful in many cases without doing the filtering. The authors could emphasize in the abstract and introduction that the system was based on the classification of novel junctions and novel splice sites, and in addition to the filtering function, the classification system would be useful."

The basis for this comment is incorrect as portcullis does not use the comparison to a reference splice site / junction to determine false junctions, rather we calculate a number of features about each junction and use these to create an initial positive and negative training set, a random forest approach is used to generate a trained model which is then used to assign a probability score to each junction. This is covered in detail in the methods section of the manuscript. We believe the confusion originates from the analysis we presented specifically figure 6 where we assess our tool on real datasets, as no ground truth dataset is available we classify junctions in to four classes based on the number of splice sites found in the reference annotation and use this to highlight that the main variation between aligners is due to the class 4 junctions. As it is not correct that the tool uses the reference annotation in the manner the reviewer indicated we have not updated the text. Portcullis does included a tool junctools that allows comparisons to be made to a reference set of junctions and we reference this in the manuscript.

2. The simulation procedure could favor the proposed filtering system since all RNA-seq reads were simulated from annotation, which is different from real RNA-seq data in which many reads are generated from unannotated junctions or splice sites. The authors could elaborate more on this limitation in the discussion.

As discussed above the reviewers question arises partly from a misunderstanding of how Portcullis identifies false junctions, as we do use the reference annotation to determine this there is no "favouring" as the reviewer might be envisaging. We do agree that simulated reads may not capture the true complexity of real data, we make this point in the "Analysis of real data" section and it is the reason why we evaluated our tool on both simulated and real data. As we have a section dedicated to analysis of real data and have described our methodology. We don't think further comment is necessary in the discussion.

Close
