# Supplementary material for: Efficient and accurate detection of splice junctions from RNA-seq with Portcullis
Source: Gigascience. 2018 Dec 12;7(12):giy131. doi: 10.1093/gigascience/giy131 (PMC6302956; doi:10.1093/gigascience/giy131)
Supplement: Reviewer-1-Report-(Original-Submission) -- Pär Engström — 5/10/2018 Reviewed [file giy131_reviewer-1-report-(original-submission).pdf]

**Reviewer Report**

**Title:** **Efficient and accurate detection of splice junctions from RNA-Seq with Portcullis**

**Version:** **Original Submission** **Date:** 5/10/2018

**Reviewer name:** **Pär Engström**

**Reviewer Comments to Author:**

This manuscript presents an analysis of the accuracy intron calls from RNA-seq read mapping tools, and describes new program (Portcullis) for improving accuracy by filtering the intron sets reported by mappers. The program seems to perform well and could be of quite wide utility, and the paper is clearly written. I have a few concerns relating to technical, but fundamental, aspects of the study.

Major points:

1. Newer versions of the alignment programs should be used. The STAR, GSNAP and HISAT2 versions used are from 2016. For all three programs, there are later releases from 2017, and for STAR and GSNAP also from 2018. Even if the study might have been initiated in 2016, it should be straightforward to rerun the analyses with updated software versions, to confirm that the results are current. The TopHat version is also not the most recent, but that is less relevant since TopHat has been superseded by HISAT2.

2. For this type of analysis, STAR should be run in 2-pass mode, since that makes it more accurate with regards to splice junctions. It would be good to check if GSNAP now has such a mode as well (last time I used GSNAP it didn't, but that was several years ago). HISAT2 uses a 2-pass strategy by default.

Minor points:

1. Page 4: "Furthermore, we can improve F1 even more by running HISAT2 in a two pass configuration, feeding in portcullis predicted junctions in the second pass." For fairness, it would be good to mention that this strategy isn't limited to HISAT2, but could be used with any mapper that accepts a list of known junctions as input (including STAR and GSNAP).

2. Page 10: "To show that these additional features…". Which are the additional features referred to here? Please clarify. The preceding paragraphs don't seem to distinguish between additional and other features.

3. Page 10: "The left column (showing results without extracted features) has more overlap be tween genuine and invalid junctions than the middle column (with extracted features)." I don't think differences in overlap are apparent from the figure. Due to the many overlapping data points, the extent of separation between classes is difficult to assess visually. Could this be presented in some other way? Could the overlap (or separation) be quantified?

4. Please specify the options used for each mapper in Methods or Supplement.

5. If I were to review a revised version, I would appreciate if a larger font was used..

**Level of Interest**

Please indicate how interesting you found the manuscript: Choose an item.

**Quality of Written English**

Please indicate the quality of language in the manuscript: Choose an item.

**Declaration of Competing Interests**

Please complete a declaration of competing interests, considering the following questions:

- Have you in the past five years received reimbursements, fees, funding, or salary from an organisation that may in any way gain or lose financially from the publication of this manuscript, either now or in the future?
- Do you hold any stocks or shares in an organisation that may in any way gain or lose financially from the publication of this manuscript, either now or in the future?
- Do you hold or are you currently applying for any patents relating to the content of the manuscript?
- Have you received reimbursements, fees, funding, or salary from an organization that holds or has applied for patents relating to the content of the manuscript?
- Do you have any other financial competing interests?
- Do you have any non-financial competing interests in relation to this paper?

If you can answer no to all of the above, write 'I declare that I have no competing interests' below. If your reply is yes to any, please give details below.

I declare that I have no competing interests

I agree to the open peer review policy of the journal. I understand that my name will be included on my report to the authors and, if the manuscript is accepted for publication, my named report including any attachments I upload will be posted on the website along with the authors' responses. I agree for my report to be made available under an Open Access Creative Commons CC-BY license (http://creativecommons.org/licenses/by/4.0/). I understand that any comments which I do not wish to be included in my named report can be included as confidential comments to the editors, which will not be published.

I agree to the open peer review policy of the journal

To further support our reviewers, we have joined with Publons, where you can gain additional credit to further highlight your hard work (see: https://publons.com/journal/530/gigascience). On publication of this paper, your review will be automatically added to Publons, you can then choose whether or not to claim your Publons credit. I understand this statement. Yes
