# Supplementary material for: Efficient and accurate detection of splice junctions from RNA-seq with Portcullis
Source: Gigascience. 2018 Dec 12;7(12):giy131. doi: 10.1093/gigascience/giy131 (PMC6302956; doi:10.1093/gigascience/giy131)
Supplement: Reviewer-2-Report-(Original-Submission) -- Shihao Shen — 17/9/2018 Reviewed [file giy131_reviewer-2-report-(original-submission).pdf]

Reviewer Report

Title: Efficient and accurate detection of splice junctions from RNA-Seq with Portcullis

Version: Original Submission Date: 5/23/2018

Reviewer name: Shihao Shen

Reviewer Comments to Author:

The authors introduced their methods for the accurate detection of splicing junction from RNA-seq data. The proposed method was based on the classification of splicing junctions into multiple categories of different confidentialities. The categories were based on the novelty of the splicing junction (i.e. whether the junction is annotated in the gene transcript annotation file) and novelty of the splice site (i.e. whether the splice site is annotated).

The classification of splicing junctions is very useful to the field of RNA-seq analysis. It is expected that junctions with novel sites are less reliable than junctions with known sites. However, it would be too arbitrary to simply filter these junctions, especially in human subjects, since many novel events are associated with disease development.

Major comments:

1. The classification-based filtering system could be too stringent for some studies, although the classification itself is useful in many cases without doing the filtering. The authors could emphasize in the abstract and introduction that the system was based on the classification of novel junctions and novel splice sites, and in addition to the filtering function, the classification system would be useful.

2. The simulation procedure could favor the proposed filtering system since all RNA-seq reads were simulated from annotation, which is different from real RNA-seq data in which many reads are generated from unannotated junctions or splice sites. The authors could elaborate more on this limitation in the discussion.

Level of Interest

Please indicate how interesting you found the manuscript: Choose an item.

Quality of Written English

Please indicate the quality of language in the manuscript: Choose an item.

Declaration of Competing Interests

Please complete a declaration of competing interests, considering the following questions:

- Have you in the past five years received reimbursements, fees, funding, or salary from an organisation that may in any way gain or lose financially from the publication of this manuscript, either now or in the future?
- Do you hold any stocks or shares in an organisation that may in any way gain or lose financially from the publication of this manuscript, either now or in the future?

- Do you hold or are you currently applying for any patents relating to the content of the manuscript?
- Have you received reimbursements, fees, funding, or salary from an organization that holds or has applied for patents relating to the content of the manuscript?
- Do you have any other financial competing interests?
- Do you have any non-financial competing interests in relation to this paper?

If you can answer no to all of the above, write 'I declare that I have no competing interests' below. If your reply is yes to any, please give details below.

I declare that I have no competing interests

I agree to the open peer review policy of the journal. I understand that my name will be included on my report to the authors and, if the manuscript is accepted for publication, my named report including any attachments I upload will be posted on the website along with the authors' responses. I agree for my report to be made available under an Open Access Creative Commons CC-BY license (http://creativecommons.org/licenses/by/4.0/). I understand that any comments which I do not wish to be included in my named report can be included as confidential comments to the editors, which will not be published.

I agree to the open peer review policy of the journal

To further support our reviewers, we have joined with Publons, where you can gain additional credit to further highlight your hard work (see: https://publons.com/journal/530/gigascience). On publication of this paper, your review will be automatically added to Publons, you can then choose whether or not to claim your Publons credit. I understand this statement. Yes
